# Corticotropin-releasing hormone (CRH) alters mitochondrial morphology and function by activating the NF-kB-DRP1 axis in hippocampal neurons

**DOI:** 10.1038/s41419-020-03204-3

**Published:** 2020-11-23

**Authors:** Chiara R. Battaglia, Silvia Cursano, Enrico Calzia, Alberto Catanese, Tobias M. Boeckers

**Affiliations:** 1 Institute of Anatomy and Cell Biology, Ulm University, Ulm, Germany; 2 International Graduate School, Ulm University, Ulm, Germany; 3 Institute for Anesthesiologic Pathophysiology and Process Engineering, Ulm University, Ulm, Germany; 4 DZNE, Ulm site, Ulm, Germany

**Keywords:** Mechanisms of disease, Stress and resilience

## Abstract

Neuronal stress-adaptation combines multiple molecular responses. We have previously reported that thorax trauma induces a transient loss of hippocampal excitatory synapses mediated by the local release of the stress-related hormone corticotropin-releasing hormone (CRH). Since a physiological synaptic activity relies also on mitochondrial functionality, we investigated the direct involvement of mitochondria in the (mal)-adaptive changes induced by the activation of neuronal CRH receptors 1 (CRHR1). We observed, in vivo and in vitro, a significant shift of mitochondrial dynamics towards fission, which correlated with increased swollen mitochondria and aberrant *cristae*. These morphological changes, which are associated with increased NF-kB activity and nitric oxide concentrations, correlated with a pronounced reduction of mitochondrial activity. However, ATP availability was unaltered, suggesting that neurons maintain a physiological energy metabolism to preserve them from apoptosis under CRH exposure. Our findings demonstrate that stress-induced CRHR1 activation leads to strong, but reversible, modifications of mitochondrial dynamics and morphology. These alterations are accompanied by bioenergetic defects and the reduction of neuronal activity, which are linked to increased intracellular oxidative stress, and to the activation of the NF-kB/c-Abl/DRP1 axis.

## Introduction

Neurons rely on mitochondria for the preservation of the membrane potential, energy supply (ATP), Ca^2+^ homeostasis, metabolite production, and ROS regulation^1–3^. Mitochondria are distributed throughout the entire neuron and they are extremely dynamic organelles, whose shape and distribution are mainly governed by two processes: fusion and fission^4,5^. A group of dynamin-related GTPases maintains the balance between these two processes, critical for the function of these organelles^5^. The Dynamin-1-like protein (DRP1) is a GTPase that functions as key regulator of mitochondrial fission^5,6^, and is recruited by the mitochondrial fission 1 protein (FIS1) to the mitochondrial outer membrane upon phosphorylation^7^. On the other hand, Mitofusins 1, 2 (MFN1, 2) and OPA1 mediate the fusion of the outer and inner membrane, respectively^8,9^.

Since synapses have high energy demands^10–12^, and despite neurons may rely on alternative ways to produce the energy necessary to sustain the presynaptic vesicle cycle^13^, neuronal mitochondria are essential for the maintenance of neurotransmission as they supply the energy sources required for neurotransmitter-containing synaptic vesicle exocytosis^14^, and plasticity. Consequently, mitochondrial defects alter neuronal plasticity, metabolism, and survival in several pathological conditions^15–18^, and reduced ATP production by mitochondria is a well characterized signal inducing neuronal apoptosis, often observed in neurodegenerative diseases^19^. In fact, pathogenic mutations in the mitochondria-related genes PINK1 and PARKIN lead to the accumulation of dysfunctional mitochondria in Parkinson’s disease^20,21^, while reduced complex II and III activity has been observed in cases of Huntington’s disease^22–24^.

Mitochondria contribute also to neuronal stress-adaptation triggered by several stress mediator molecules, such as corticotropin-releasing hormone (CRH, mainly produced in the hypothalamus^25^), glucocorticoids (GCs, lipophilic hormones produced within the adrenal cortex and reaching the brain by passing through the blood-brain barrier^25^), adrenocorticotropin (ACTH, released from the pituitary gland^25^), and catecholamines (mainly produced within the adrenal medulla, but also within the brain, such as dopamine^26,27^). Their response to these stress stimuli consists of morphological and functional modifications as (i) fusions/fission dynamic changes; (ii) reactive oxygen species (ROS) production; (iii) hormonal receptors activation; (iv) potential mtDNA damage; (v) energy capacity alteration and (vi) production of signaling molecules (mitokines) regulating cellular physiology^28^. Indeed, mitochondrial membrane potential alterations have been reported in stress-induced signaling in post-traumatic stress disorders^29,30^, while DRP1-dependent mitochondrial alterations have been associated with dramatic memory impairments in a traumatic brain injury animal model^31^.

Our group has described a dramatic (but reversible) CRH-dependent loss of hippocampal synapses and cognition impairment (in absence of neuronal death) after blunt thorax trauma (TxT)^32^, which opened the question whether mitochondria might also be directly involved in the maladaptive alterations triggered by the activation of the CRH receptor 1 (CRHR1). To answer this, we investigated the effect of CRH on mitochondrial dynamics, structural organization and functionality in hippocampal neurons. We show that CRHR1 activation triggers a profound remodeling of the mitochondrial network and bioenergetics properties, which depend on the activation of the NF-kB/DRP1 axis. To our surprise, these alterations occurred in a context of preserved ATP production, that might explain the spontaneous neuronal recovery upon long-term CRH treatment.

## Materials and methods

### Animal housing and ethics statement

Animals were purchased from Janvier Labs. Male C57BL/6JRj mice (8–10-week old, body weight 25 ± 1.5 g) were group-housed, while pregnant female Sprague-Dawley rats were housed alone in a single cage for 5 days after delivery until dissection was performed. All animals were housed on a 12/12-h light/dark cycle (light on at 7:00 AM), with ad libitum access to food and water. All animal experiments in this study were approved by the review board of the Land Baden-Württemberg (Permit Numbers: O.103 and 1233) and performed in compliance with the ARRIVE Guidelines and with the guidelines for the welfare of experimental animals issued by the Federal Government of Germany and the Max Planck Society.

### Thorax trauma

To perform TxT, mice were anesthetized with a mixture of 2.5% sevoflurane (SevoraneTM, Abbott, Wiesbaden, Germany) and 97.5% oxygen at a continuous flow of 0.5 L/min and a FiO2 of 1.0. The mice were fixed to an acrylic glass plate in the supine position, and the abdomen and chest were shaved. Before termination of anesthesia, buprenorphine (0.03 mg per kg body weight) was injected subcutaneously to provide suitable analgesia. TxT was induced by a single blast wave centered on the thorax as previously described by Cursano et al.^32^. One control group (Sham) of animals were subjected to the same experimental procedure, without TxT. The analysis was performed 5 days post injury; at this time point, animals were sacrificed and tissues collected for biochemical investigations.

### Primary rat hippocampal neurons

Primary cultures of rat hippocampal neurons were prepared from embryos at E17-E18, as previously described in Catanese et al.^33^. In brief, embryonic brains were dissected out and placed in Hanks’ Balanced Salt Solution w/ CaCl_2_ w/ MgCl_2_ (HBSS, Gibco) at 4 °C; hippocampi were manually dissected under stereomicroscopic guidance. The tissues were incubated for 15 min with 0.25% trypsin-EDTA (1x) (Gibco) at 37 °C and 5% CO_2_ under gentle shakings. After one wash with Dulbecco’s Modified Eagle Medium-high glucose (4.5 g/L) (DMEM, Gibco), supplemented with 10% fetal bovine serum (FBS, Sigma), 1% penicillin/streptomycin (P/S, Gibco) and 1% GlutaMAX (100x, Gibco), the tissues were mechanically dissociated in Neurobasal medium (1x, Gibco) supplemented with 2% B27 (50x, Gibco), 1% GlutaMAX and 1% P/S at 100 U/ml (Invitrogen) (henceforth NB^+^ medium) and the cells were filtered using a 100 µm mesh filter, and resuspended in NB^+^. The hippocampal neurons were then plated on coverslips coated with poly-l-lysine 0.05–0.1 mg/ml (Sigma–Aldrich, Germany) on Petri dishes (100 × 20 mm), 6- or 24-well plates. Cells were maintained in Neurobasal medium supplemented with 2% B27, 1% GlutaMAX and 1% P/S at 100 U/ml at 37 °C in a humidified atmosphere containing 5% CO_2_; medium was half-renewed weekly. For transmission electron microscopy (TEM), cells were plated on sapphire discs coated with carbon (using a BAF 300 electron beam evaporation device) (Balzers) followed by poly-L-lysine coating, and samples were fixed by high-pressure freezing as previously described^33^. All the experiments and treatments were performed at day in vitro 14 (DIV14).

The following chemicals were used in the study: CRH peptide (Bachem #H-2435, stock solution 100 μM in water); CRH receptor blocker NBI30775 (Hycultec GmbH, #HY-14127, final concentration 10 μg/ml in sterile DMSO); Imatinib mesylate, c-Abl inhibitor (Abcam; #ab142070, final concentration 3 μM in DMSO); JSH-23 (Abcam, #ab144824, final concentration 10 μM in DMSO) to inhibit NF-κB nuclear translocation; CNQX disodium salt (Abcam, #ab120044, final concentration 10 μM in water) to block the AMPA/kainate activity. In order to reduce the stress to cultured neurons, and to provide them with the neurotrophic factors produced by non-neuronal cells^34^, we did not treat our cultures with mitosis inhibitors. This led to neuron-glia mixed cultures, which at DIV 14 contained 70% of neurons and 30% of astrocytes (Fig. Suppl. 1A). Of note, the 92% of all neurons in culture were excitatory (VGlut1 positive), while only the 8% was positively stained against the specific inhibitory marker GAD67 (Fig. Suppl. 1B).

### Antibodies list

The primary antibodies used in this study are listed in Table 1. We used secondary antibodies coupled to Alexa Fluor® 488, 568 or 647 (all from Life Technologies, dilution 1:500) for immunocytochemistry, and HRP-conjugated for western blot (Dako, Glostrup, Denmark, dilution 1:1000).

**Table 1:** List of the primary antibodies used.

Identification	Company	Article no.	Technique	Dilution
Map2	EnCorBiotechology Inc.	CPCA-MAP2	ICC	1:500
Vglut1	SynapticSystems GmbH	135304	ICC	1:500
Shank2	In house	ppI-SAM157 pabSA5192	ICC	1:500
cAbl	Biorbyt	orb156228	ICC	1:500
TOMM20	Abcam	ab56783	ICC	1:1000
Gfap	Synaptic System	173011	ICC	1:1000
Gad67	Abcam	ab213508	ICC	1:1000
iNOS	ThermoFisher	PA1-036	WB	1:1000
Cyt c	BD Bioscience	556432	ICC	1:1000
IL-6	Cell Signaling	129125	WB	1:1000
IL-17	Abcam	ab79656	WB	1:1000
LC3A	Cell Signaling	4599	ICC	1:1000
Creb^S133^	Abcam	ab32096	WB	1:1000
Creb	Abcam	ab32515	WB	1:1000
Synaptotagmin	Synaptic System	105311C3	ICC	1:500
DRP1	Abcam	ab184247	WB	1:1000
DRP1^S616^	Cell Signaling	3455	WB	1:1000
Mitofusin 1	Abcam	ab104274	WB	1:1000
Mitofusin 2	Abcam	ab56889	WB	1:1000
OPA1	Abcam	ab42364	WB	1:1000
Actin	Sigma-Aldrich	#A2228	WB	1:250000

### Western blot

Ten micrograms of proteins were loaded onto 10% SDS-PAGE and western blot experiments performed as previously described^33^. For quantification of protein levels, Gel-analyzer Software 2010a was used.

### Immunohistochemistry

Animals were anesthetized with a mixture of 25% ketamine and 5% xylazine solubilized in a NaCl solution, and perfused using 25 ml of cooled PBS and 50 ml of 4% PFA. Then, the brains were treated as previously described by Heise et al.^35^. In brief, brains were incubated in 4% PFA overnight and then left in 30% sucrose for 24 hours. Next, brains were frozen in OCT and kept at −80 °C until cryostat cutting. Brains cutting was performed at −20 °C using a cryostat (Leica CM3050 S) and appropriate microtome blades (Feather, A35 Type). Brain sections were put in PBS^−/−^ for free-floating antibody labeling. First, sections were left in blocking solution (3% BSA + 0.3% Triton-X-100, diluted in PBS^−/−^) for 2 h at RT on the horizontal shaker; afterwards, sections were incubated with primary antibody (prepared in the blocking solution) for 48 h at 4 °C. After three washes in PBS^−/−^ and the secondary antibody incubation for 2 h at RT (antibodies coupled to Alexa Fluor® 488, 568 or 647 (all from Life Technologies), brain sections were mounted using VectaMount (Vector labs) containing 4′,6-diamidino-2-phenylindole (DAPI). Confocal microscopy was performed with a laser-scanning microscope (Leica DMi8) equipped with an ACS APO 63x oil DIC immersion objective. Images were acquired using the LasX software (Leica), with a resolution of 1024 × 1024 pixels and a Z-stacks of 6.5 μm (step size of 14 × 0.5).

### Immunocytochemistry

Immunocytochemistry was performed as described previously^33^. Cells were fixed for 5 min in 4% paraformaldehyde (PFA), permeabilized and blocked in PBS^−/−^ added with 10% Goat serum and 0.2% Triton-X-100, and thereafter incubated with primary antibodies for 48 h at 4 °C. after incubation, cells were washed three times for 30 min in PBS^−/−^ and then incubated with the secondary antibodies for 2 h at room temperature. Cells were again washed three times for 30 min in PBS^−/−^ before mounting with VectaMount (Vector labs) containing DAPI onto microscope glass slides.

### Fluorescence microscopy and image analysis

In this study, we used an upright fluorescence microscope (Zeiss Axioskop 2), equipped with an Axiocam 506 mono camera, and a Plan-Neofluar 20× air or Plan-Neofluar 40x oil immersion objective. The Axiovison 4.7.1 software (Zeiss, Germany) was used for image acquisition. For the analysis of hippocampal excitatory synapses, three different dendrites of three different neurons acquired from three different wells were analyzed for each condition in each independent experiment, using Bitplane Imaris software.

### Synaptotagmin assay

Primary neurons were incubated with an antibody raised against the luminal tail of synaptotagmin-1 (1:500) for 30 min together with CRH (with or without antagonist). Then, DIV14 neurons from all the experimental groups were fixed for ICC. At active synapse, neurotransmitters are released by calcium-triggered synaptic vesicle exocytosis^36^ that is mediated by synaptotagmins 1 and 2^37^. Thus, this assay allows the quantification of active synapses positively labeled.

### Multi-electrode array (MEA) measurements

We employed a MaxTwo Multiwell MEA system (MAXWELL Biosystems) to investigate the effect of CRH on neuronal activity. Recordings were carried at DIV 14: activity was measured by performing an Activity Scan Assay by recording the full electrode chip. Only action potentials reaching a spike threshold of 5 above background noise were recorded and used for analysis. After the first scan, the same cells were treated either with CRH or with CRH + NBI for 30 min, before performing the Activity Scan again. Results were obtained by comparing the same cultures before and after the different treatments. Data were obtained from six wells for each treatment conditions derived from two independent replicates.

### Dendritic degeneration index

Dendritic degeneration was analyzed by immunostaining for MAP2. Primary hippocampal neurons at DIV14 were incubated with CNQX disodium salt (Abcam, #ab120044, final concentration 10 μM in water) for 30 min to block the AMPA/kainate activity in resting conditions and in combination with CRH (100 nM). Images were acquired with a ×40 objective lens using a Zeiss Axioskop 2 microscope and analyzed with the ImageJ software. We detect the degenerated dendrites as described in Yuva-Aydemir et al.^38^ with minor changes. In brief, we used the particle analyzer module of ImageJ on binarized images to calculate the area of the small fragments or particles (size 10–infinity pixels). The dendritic degeneration index was defined as the ratio of the area of degenerated dendrites to the total dendrite area (healthy plus degenerated dendrites). Three different dendrites of three different neurons acquired from three different wells were analyzed for each condition in each independent experiment. In the graph, each independent data point represents one preparation (*n* = 3 independent cultures).

### TEM quantitative analysis

TEM images were acquired in a Jeol JEM 1400 transmission electron microscope at 120 kV. A magnification of ×40000 was chosen to study the mitochondrial ultrastructure within an image. ImageJ software was used to determine the area of the mitochondria and the number of mitochondria was manually counted (field of view: 12.77 μm^2^). Mitochondria were classified regarding their shape as: rod, swollen or irregular shapes^39–42^. Moreover, the mitochondrial *cristae* were investigated and distinguished in well-defined *cristae* and aberrant *cristae*^43–45^.

### High-resolution respirometry procedures

**Fig. 1:**
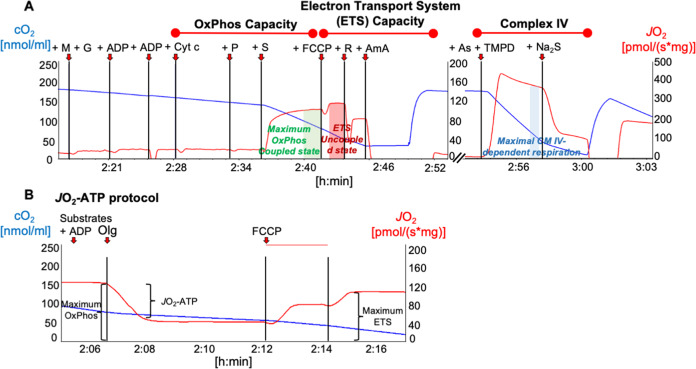
Representative high-resolution respirometry recordings of primary hippocampal neurons. The oxygen flux (*J*O_2_, red line) is calculated as the negative slope of the oxygen concentration (cO_2_, blue line) with time (*x*-axis), normalized for cell number, and corrected for oxygen back-diffusion. Red arrows indicate times of injection of substrates and inhibitors. **A** The protocol for the evaluation of OxPhos capacity, ETS capacity, and Complex IV activity includes the following steps (final chamber concentrations): co-injection of malate (2 mM) and glutamate (10 mM), ADP (4.5 mM) and pyruvate (5 mM), cytochrome c (10 µM) (test for mitochondrial outer membrane integrity) and succinate (10 mM, OxPhos-capacity), FCCP (0.5 mM, ETS-capacity), rotenone (0.5 µM) antimycin A (5 µM), co-injection of ascorbate (2 mM) and TMPD (0.5 mM), Na_2_S (40 µM) (Complex IV activity). **B** A slight modification of this protocol allowed to determine the O_2_ consumption related to ATP production (*J*O_2_ATP) in a separate series of experiments. This modification consisted of an additional injection of the ATP-synthase inhibitor oligomycin (2.5 μM) between succinate and FCCP. All other steps of the protocol were maintained unchanged. The *J*O_2_ATP was calculated as the *J*O_2_-difference before and after oligomycin.

Mitochondrial respiration was quantified by high-resolution respirometry (HRR) using the Oroboros Oxygraph-2K (Oroboros Instruments, Innsbruck, Austria). Cultured neurons were harvested, centrifuged at 1500 × *g* for 5 min at 37 °C, and suspended in 1200 μL of respiration buffer containing 0.5 mM EGTA, 3 mM MgCl_2_·6H_2_O, 60 mM Lactobionic acid, 20 mM Taurine, 10 mM KH_2_PO_4_, 20 mM HEPES, 110 mM Sucrose, 1 g L^−1^ bovine serum albumin. One milliliter of each sample were added to the oxygraph chambers. Mitochondrial respiration was quantified in terms of oxygen flux (*J*O_2_) based on the rate of change of the O_2_ concentration in the chambers after normalization to the total cell number: pmol O_2_/(s 10^6^ cells). By sequential addition of substrates (10 mM glutamate, 2 mM malate, 5 mM pyruvate, and 10 mM succinate) and ADP (5 mM) the maximum mitochondrial respiration in the coupled state was achieved (maximum OxPhos) (Fig. 1A). Cytochrome *c* (Cyt c) 10 μM was added in an intermediate step after ADP to check for mitochondrial outer membrane integrity; an eventual damage would be indicated by an increase in *J*O_2_ in response to Cyt c. Maximum mitochondrial respiration in the uncoupled state (maximum electron transport system—ETS) was evaluated as the next step after the achievement of the maximum OxPhos condition by further addition of the uncoupling agent Carbonyl cyanide-4-(trifluoromethoxy)-phenylhydrazone (FCCP, final concentration 0.5 mM). Finally, cytochrome c oxidase (cCOX) activity was measured after inhibition of complex I and complex III by rotenone 0.5 μM and antimycin A 5 μM respectively, by addition of 2 mM ascorbate and 0.5 mM tetramethylphenylendiamine (TMPD) (Fig. 1A). Since TMPD is subject to auto-oxidation, cCOX-dependent respiration is calculated as previously described^46^ by subtracting the residual *J*O_2_ remaining after the addition of 40 µM of the cCOX-inhibitor sodium azide (Na_2_S) from the maximum *J*O_2_ previously achieved immediately after the injection of TMPD.

The *J*O_2_ related to ATP production (*J*O_2_-ATP) was measured in a separate series of experiments by adding a step consisting in the injection of 1.25 µM of the ATP-synthase inhibitor oligomycine between the maximum OxPhos and the maximum ETS condition (Fig. 1B). *J*O_2_-ATP was calculated as the difference between the respiration rate before and after the addition of oligomycine. Assuming a constant ATP/O ratio, the *J*O_2_-ATP was used as an indicator of ATP-production. However, the ATP/O ratio cannot be assumed as a constant, and it is not known in our experiment. We therefore prefer to avoid speculating on ATP-production based on this experimental sequence.

### Mitochondrial network labeling and analysis

Neuronal mitochondria were labeled with MitoTracker™ Red CMXRos (100 nM final concentration, Invitrogen-Molecular Probes #M7512) for 30 min at 37 °C. The neuronal cultures were then fixed in 4% PFA for 5 min at 37 °C and imaged. For the analysis, we used the mitochondrial network analysis (MiNA) toolset, an ImageJ macro which allows the semiautomated analysis of mitochondrial network, providing a topological skeleton of mitochondria^47^. Three parameters were considered to describe the mitochondrial network complexity: mitochondrial footprint (or volume), summed branch lengths mean and network branches mean.

### Mitochondrial network labeling and analysis in vivo

MiNA was performed by Bitplane Imaris software, using the surface tool. Three regions of interest (ROIs), containing an average of four cells (DAPI signal), were selected for each image and the surface of the mitochondrial network was detected (Cyt c signal) within the ROIs. We obtained the mitochondrial network masks in which each mitochondrial network is labeled by one different color. We classified the networks according to their area: mitochondrial network with area smaller or equal to 10 μm^2^, between 10 and 200 μm^2^ and greater or equal to 200 μm^2^. The larger the area, the higher the mitochondrial network complexity.

### S-Nitrosylation assay

The assay was performed using the Pierce™ S-Nitrosylation Western Blot-Kit (Thermo Scientific™) following the manufacturer’s protocol. In brief, cell lysates were treated with ascorbate in HENS buffer for specific labeling with iodoTMT reagent after MMT pretreatment (Thermo Scientific™, #23011). Protein labeling was confirmed by Western blot using TMT antibody (Thermo Scientific™, #90075), according to standard procedures.

### Statistical analyses

Data are displayed as mean ± SEM, and those related to immunostainings and immunoblots are expressed as fold change of the respective control group. For statistical analysis, we tested normally distributed data by using *t*-Test and one-way ANOVA followed by Bonferroni’s post hoc multiple comparison test, while non-normally distributed data were analyzed with the Kruskal–Wallis non parametric test combined with uncorrected Dunn’s multiple comparison test. Statistical analysis was performed using GraphPad Prism (Version 7.0). Significance was set at *p* < 0.05.

## Results

### CRH alters mitochondrial network and morphology

**Fig. 2:**
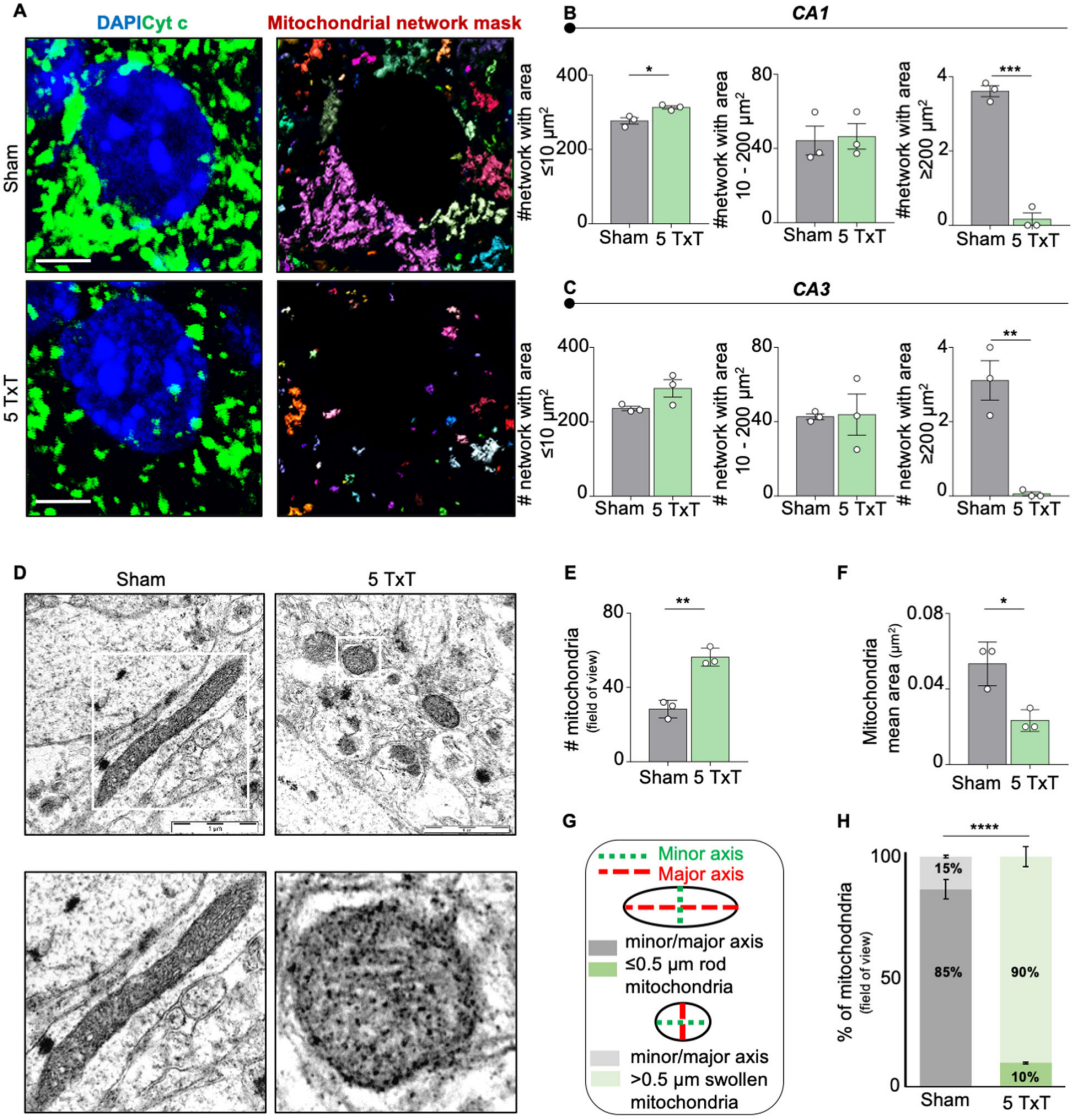
Thorax trauma (TxT) mouse model shows mitochondrial fragmentation in the CA1 region of the hippocampus. **A** IHC to detect somatic mitochondrial network complexity in hippocampal CA1 and CA3 regions using Cyt c (green) and DAPI (blu) (scale bar = 5 μm) in Sham and in mice 5 days after TxT (5 TxT); mitochondrial network mask panel shows each mitochondrial structure labeled by one different color. **B** Quantification of the number (#) of somatic mitochondrial networks with area: ≤10 μm^2^; between 10 and 200 μm^2^; ≥200 μm^2^ in the CA1 (stratum radiatum) and **C** CA3 (stratum lucidum) regions. Three ROIs, containing an average of 4 neurons (DAPI signal), were selected for each image and the surface of the mitochondrial network was detected (Cyt c signal) within the ROIs. **D** Representative electron microscopy (EM) images of CA1 somatic hippocampal mitochondria (Sham vs 5 TxT) (scale bar = 1 μm). All the mitochondria per field of view were considered. **E** Quantification of number (#) of mitochondria and **F** mitochondrial mean area/12.77 μm^2^ (μm^2^). **G** Mitochondrial shapes categories: mitochondria with a minor/major ratio ≤0.5 μm are rod-shaped; mitochondria with a minor/major ratio >0.5 μm are swollen-shaped; and **H** relative quantification. Experiments were performed in *N* = 3 independent replicates at DIV14. Data are displayed as Mean ± SEM; one-way ANOVA and Bonferroni’s post hoc comparison test, or exact Fisher´s test in **H** were performed (*p < 0.05, **p < 0.005, ***p < 0.0005, ****p < 0.0001).

Since CRH triggers a dramatic loss of synapses^32^, which require high amount of energy (ATP)^48^, we investigated the effect of CRH release on neuronal mitochondria by analysing their morphology in mice 5 days after trauma (5 TxT) (Fig. 2A). We found a significant increase in the number of mitochondrial networks with an area ≤10 μm^2^ and a simultaneous reduction of networks larger than 200 μm^2^ in the hippocampal CA1 (# network with area ≤10 μm^2^: 276.8 ± 8.623 μm^2^ in Sham vs 313.4 ± 4.314 μm^2^ in 5 TxT, *p* = 0.0191; # network with area 10–200 μm^2^: 44.22 ± 7.752 μm^2^ in Sham vs 46.44 ± 6.838 μm^2^ in 5 TxT, *p* = 0.8403; # network with area ≥200 μm^2^: 3.611 ± 0.147 μm^2^ in Sham vs 0.1667 ± 0.1667 μm^2^ in 5 TxT, *p* = 0.0001; Fig. 2B) and CA3 (# network with area ≤10 μm^2^: 235.9 ± 5.877 μm^2^ in Sham vs 290 ± 23.66 μm^2^ in 5 TxT, *p* = 0.0907; # network with area 10–200 μm^2^: 42.72 ± 1.544 μm^2^ in Sham vs 43.83 ± 11.07 μm^2^ in 5 TxT, *p* = 0.9256; # network with area ≥200 μm^2^: 3.111 ± 0.53 μm^2^ in Sham *vs* 0.0555 ± 0.0555 μm^2^ in 5 TxT, *p* = 0.0046; Fig. 2C) regions of TxT animals. These alterations were confirmed by TEM analysis (Fig. 2D), which highlighted a significant larger number of mitochondria (# mitochondria: 28.33 ± 2.728 in Sham vs 56.33 ± 2.848 in 5 TxT, *p* = 0.0021; Fig. 2E), whose area was significantly smaller (mitochondria mean area: 0.05 ± 0.006 μm^2^ in Sham vs 0.02 ± 0.003 μm^2^ in 5 TxT, *p* = 0.0158; Fig. 2F) in the TxT group when compared to Sham animals. In addition, TxT significantly increased the percentage of swollen (ratio between major and minor axis larger than 0.5 μm) mitochondria (a proxy for mitochondrial dysfunction^35,36^) (percentage of mitochondria with minor/major axis ≤0.5 μm: 85.84 ± 7.172% in Sham vs 10.43 ± 3.653% in 5 TxT, *p* = 0.0007; percentage of mitochondria with minor/major axis >0.5 μm: 14.16 ± 7.172% in Sham vs 89.57 ± 3.653% in 5 TxT, *p* = 0.0007; Fig. 2G, H).

**Fig. 3:**
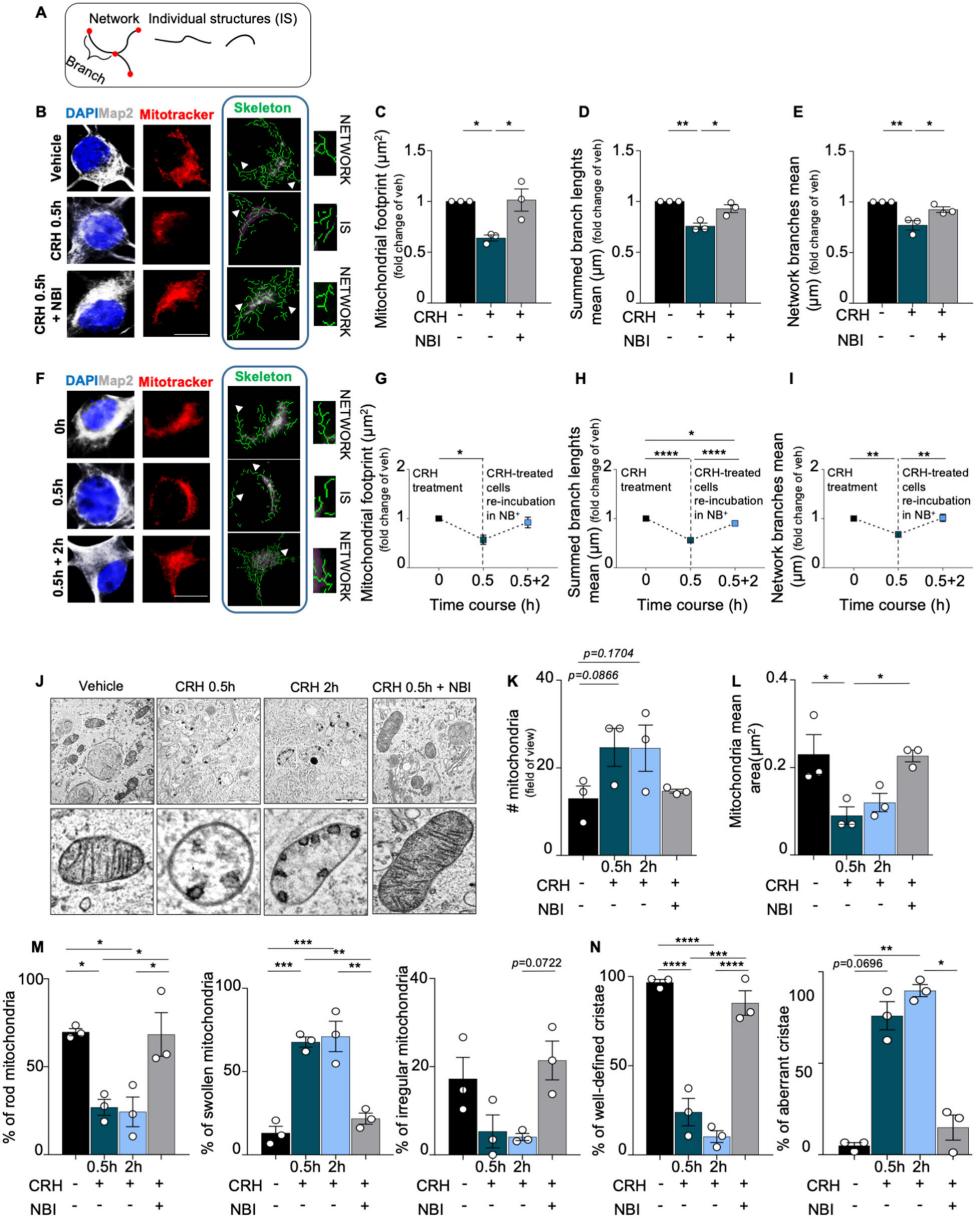
CRH controls mitochondrial cristae remodeling, swelling and network complexity in primary hippocampal neurons. **A** Schematic representation of mitochondrial structures: mitochondrial network (composed by two or more branches) and unbranched individual structures (IS). **B** Representative images of somatic mitochondrial network complexity, IHC for MAP2 (gray), mitochondria labeled with MitoTracker^TM^ (red), mitochondrial skeletonized structures (green) obtained with MiNa toolset^39^ (white arrowheads indicates examples of networks and IS) (scale bar = 5 μm) for all different experimental conditions (vehicle, CRH 100 nM 0.5 h and CRH 0.5 h + CRHR1 Blocker NBI30775 100 nM) and relative analysis: **C** mitochondrial footprint (μm), **D** summed branch lengths mean (μm) and **E** network branches mean (μm). **F** Cells were treated with CRH 100 nM for 0.5 h followed by Neurobasal Medium plus B27 for 2 h and 5 h. IHC for MAP2 (gray), mitochondria labeled with MitoTracker^TM^ (red), mitochondrial skeleton generated by Mitochondrial Network Analysis (MiNa) toolset (green)^39^ (white arrowheads indicates examples of networks and IS) (scale bar = 5 μm) and **G**–**I** relative time-course analysis of the same parameters described above. Somatic mitochondrial networks were analyzed in six neurons for each condition for each different independent preparation. **J** Representative electron microscopy (EM) images of somatic hippocampal mitochondria for all different experimental conditions (vehicle, CRH 100 nM 0.5 h and 2 h; CRH 0.5 h + CRHR1 Blocker NBI30775 100 nM) (scale bar = 1 μm). TEM images showing mitochondrial *cristae* remodeling in treated neurons. All the mitochondria per field of view were considered. **K** Quantification of number (#) of mitochondria and **L** mitochondrial mean area/12.77 μm^2^ (μm^2^). **M** Percentage of rod, swollen and irregular mitochondria. **N** Percentage of well-defined *cristae* and aberrant *cristae*. Experiments were performed in = 3 independent replicates at DIV14. Data are displayed as Mean ± SEM; one-way ANOVA and Bonferroni’s post hoc comparison test were performed; data non normally distributed were analyzed by Kruskal–Wallis non parametric test followed by Uncorrected Dunn’s multiple comparison test. (*p < 0.05, **p < 0.005, ***p < 0.0005, ****p < 0.0001).

To confirm that the mitochondrial aberrations occurring after peripheral trauma were specifically induced by CRH release (as in the case of synaptic loss; Cursano et al.^32^) we exposed primary hippocampal neurons (Fig. Suppl. 1) to the hormone (100 nM) or vehicle (DMSO) for 30 min. By imaging MitoTracker^TM^ staining and using the MiNA toolset^47^ (Fig. 3B), we found that CRH drastically reduced the mitochondrial network complexity (mitochondrial footprint: CRH 0.5 h 0.6404 ± 0.0293 μm^2^ fold of vehicle, *p* = 0.0240; Fig. 3C), the length (summed branch length mean: CRH 0.5 h 0.7583 ± 0.0300 μm fold of vehicle, *p* = 0.0026; Fig. 3D), and the number of network branches (network branches mean: CRH 0.5 h 0.7704 ± 0.0468 fold of vehicle, *p* = 0.0061; Fig. 3E). Notably, re-incubation of CRH-treated neurons with conditioned NB^+^ medium for 2 hours (Fig. 3F) normalized the alterations in all the parameters analyzed, which appeared comparable to those of vehicle-treated cells (mitochondrial footprint: CRH 0.5 h 0.5685 ± 0.0934 μm^2^ fold of vehicle, *p* = 0.0306; CRH + NB^+^ 2 h 0.9201 ± 0.1085 μm^2^ fold of vehicle, *p* > 0.9999; summed branch length mean: CRH 0.5 h 0.5594 ± 0.0236 μm fold of vehicle, *p* < 0.0001; CRH + NB^+^ 2 h 0.901 ± 0.0224 μm fold of vehicle, *p* = 0.0297; 0.5594 ± 0.0236 μm in CRH 0.5 h vs 0.901 ± 0.0224 μm in CRH + NB^+^ 2 h, *p* < 0.0001; network branches mean: CRH 0.5 h 0.6755 ± 0.03 μm fold of vehicle, *p* = 0.0080; CRH + NB^+^ 2 h 1.015 ± 0.0750 μm fold of vehicle, *p* > 0.9999; 0.6755 ± 0.03 μm in CRH 0.5 h vs 1.015 ± 0.0750 μm in CRH + NB^+^ 2 h, *p* = 0.0064; Fig. 3G-I). Moreover, co-treatment of neurons with CRH and the CRHR1 antagonist NBI30775 (henceforth NBI) completely prevented these mitochondrial alterations as well (mitochondrial footprint: CRH + NBI 1.014 ± 0.1091 μm^2^ fold of vehicle, *p* = 0.0203; summed branch length mean: CRH + NBI 0.9299 ± 0.0376 μm fold of vehicle, *p* = 0.0143; network branches mean: CRH + NBI: 0.924 ± 0.0273 fold of vehicle, *p* = 0.0399; Fig. 3C–E).

We then analyzed the ultrastructural alterations occurring in vitro upon CRH treatment. In line with the in vivo data, TEM analysis (Fig. 3J) revealed that CRH-treated cells were characterized by a larger number of mitochondria (# mitochondria: 26 ± 5.686 in vehicle vs 49.33 ± 8.667 in CRH 0.5 h, *p* = 0.0866 and vs 49 ± 10.58 in CRH 2 h, *p* = 0.1704; Fig. 3K), which were significantly smaller than in vehicle-treated ones (mitochondria mean area: 0.23 ± 0.0450 μm^2^ in vehicle vs 0.09 ± 0.02 μm^2^ in CRH 0.5 h, *p* = 0.0309; 0.2267 ± 0.0133 μm^2^ in CRH + NBI vs CRH 0.5 h, *p* = 0.0124; Fig. 3L). These morphological alterations were detectable starting from 30 minutes after incubation with CRH (analysis at earlier time points revealed a progressive tendency toward the changes later observed; Fig. Suppl. 2A, B), and increasing the exposure to CRH up to 2 h did not further aggravate the structural phenotype. Based on their structural conformation, we classified the mitochondria as “rod”, “swollen”, and “irregular”^39,40^ (Fig. Suppl. 2C): Rod—normal appearing mitochondria mostly longitudinally oriented with well-organized *cristae*; Swollen—rounded appeared mitochondria with disrupted *cristae*; Irregular—mitochondria that show irregular shapes, not referable to the rod and swollen ones. CRH treatment significantly enlarged the population of swollen mitochondria (a proxy for mitochondrial dysfunction^43,44^, while the rod and irregular ones were reduced in treated neurons (percentage of rod mitochondria: 69.72 ± 2.017% in vehicle vs 26.94 ± 4.553% in CRH 0.5 h, *p* = 0.0303 and vs 24.35 ± 8.476% in CRH 2 h, *p* = 0.0219; 68.36 ± 12.39% in CRH + NBI vs CRH 0.5 h, *p* = 0.0360 and vs CRH 2 h, *p* = 0.0259; percentage of swollen mitochondria: 13.04 ± 4.098% in vehicle vs 67.74 ± 3.028% in CRH 0.5 h, *p* = 0.0006 and vs 71.08 ± 9.111% in CRH 2 h, *p* = 0.004; 21.71 ± 3.31% in CRH + NBI vs CRH 0.5 h, *p* = 0.0021; CRH 2 h vs CRH + NBI, p = 0.0013; percentage of irregular mitochondria: 4.059 ± 0.8075% in CRH 2 h vs 21.42 ± 4.399% in CRH + NBI, *p* = 0.0722; Fig. 3M). Also in this case, the effect of CRH was seen only after 30 min, since earlier analysis highlighted comparable mitochondrial populations among treatments (Fig. Suppl. 2D). Interestingly, we detected a drastic increase in the number of mitochondria characterized by aberrant cristae already after 5 min of CRH exposure (percentage of aberrant cristae: 4.919 ± 2.465% in vehicle vs 46.13 ± 6.166% in CRH 5′, *p* = 0.0124, and vs 56.79 ± 9.063% in CRH 15′, *p* = 0.0039; Fig. Suppl. 2E), which increased until 30 min and remained stable up to 2 h of CRH (percentage of aberrant cristae: 4.981 ± 1.609 in vehicle vs 75.98 ± 7.719% in CRH 0.5 h, *p* = 0.0696, and vs 89.74 ± 3.295 in CRH 2 h, *p* = 0.0091; 89.74 ± 3.295 in CRH 2 h vs 14.84 ± 6.872 in CRH + NBI, *p* = 0.0233; Fig. 3N). Importantly, none of these alterations were detectable in the CRH + NBI treatment group, indicating a central role of CRHR1 activation in this process (# mitochondria: 26 ± 5.686 in vehicle vs 29 ± 0.8819 in CRH + NBI, *p* = 0.9091; mitochondria mean area: 0.23 ± 0.0450 μm^2^ in vehicle vs 0.2267 ± 0.0133 μm^2^ in CRH + NBI, *p* = 0.7332; percentage of rod mitochondria: 69.72 ± 2.017% in vehicle vs 68.36 ± 12.39% in CRH + NBI, *p* > 0.9999; percentage of swollen mitochondria: 13.04 ± 4.098% in vehicle vs 21.71 ± 3.31% in CRH + NBI, *p* > 0.9999; percentage of irregular mitochondria: 17.24 ± 4.879% in vehicle vs 21.42 ± 4.399% in CRH + NBI, *p* > 0.9999; percentage of aberrant cristae: 4.981 ± 1.609% in vehicle vs 14.84 ± 6.872% in CRH + NBI, *p* = 0.7336; Fig. 3K–N).

### CRH alters the activity of the mitochondrial respiratory chain, but not ATP availability

**Fig. 4:**
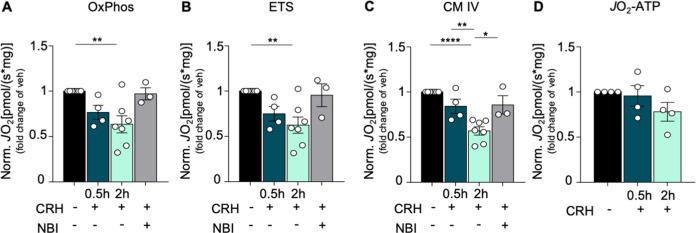
CRH alters mitochondrial respiratory activity. Mitochondrial respiratory activity of CRH-treated primary neurons (100 nM CRH for 0.5, 2 h and CRH 0.5 h + CRHR1 Blocker NBI30775 100 nM) measured by high-resolution respirometry using the Oxygraph-2k(R) system (OROBOROS Instruments Corp., Innsbruck, Austria): **A** OxPhos, maximum oxidative phosphorylation in the coupled state with complex I and II substrates, pyruvate and ADP; **B** ETS, maximum mitochondrial respiratory activity after uncoupling with FCCP; **C** CM IV, uncoupled mitochondrial respiratory activity linked to complex IV upon inhibition of complex I by rotenone; **D** *J*O_2_ATP, the ATP production-related oxygen flux. Note that *J*O_2_ was normalized to the total cell number: pmol O_2_/(s*10^6^ cells). Experiments were performed in *N* = 3–7 independent replicates at DIV14. *J*O_2_ATP experiments were performed in *N* = 4 independent replicates at DIV14. Data are displayed as Mean ± SEM; one-way ANOVA and Bonferroni’s post hoc comparison test were performed. (*p < 0.05, **p < 0.005, ***p < 0.0005, ****p < 0.0001).

Next, we performed high-resolution respirometry (HRR; representative l tracings obtained are shown in Fig. 1) to investigate whether the morphological alterations observed upon CRH treatment correlate with bioenergetic defects. Hippocampal neurons showed a significant decrease in the maximum OxPhos capacity (OxPhos: CRH 2 h 0.6347 ± 0.0938 *J*O_2_ fold of vehicle, *p* = 0.0043; Fig. 4A) as well as in the maximum ETS (ETS: CRH 2 h 0.6239 ± 0.0896 *J*O_2_ fold of vehicle, *p* = 0.0049; Fig. 4B) and in the activity of complex IV (CM IV: CRH 2 h 0.5691 ± 0.0454 *J*O_2_ fold of vehicle, *p* < 0.0001; 0.8414 ± 0.0792 *J*O_2_ in CRH 0.5 vs CRH 2 h, *p* = 0.0089; 0.8575 ± 0.1016 *J*O_2_ in CRH + NBI vs CRH 2 h, *p* = 0.0121; Fig. 4C) upon 2 h of CRH incubation, when compared to vehicle. Analysis of earlier time points of CRH incubation did not result in any differences among the treatments (Fig. Suppl. 3A-C). Co-treatment of primary neurons with CRH and the CRHR1 antagonist NBI restored the activity of the mitochondrial respiratory chain (OxPhos: CRH + NBI 0.9702 ± 0.0663 *J*O_2_ fold of vehicle, *p* > 0.9999; ETS: CRH + NBI 0.9543 ± 0.1265 *J*O_2_ fold of vehicle, *p* > 0.9999; CM IV: CRH + NBI 0.8575 ± 0.1016 *J*O_2_ fold of vehicle, *p* = 0.5389; Fig. 4A–C), which also spontaneously recovered after 5 h of CRH treatment without replacing the culture medium (data not shown). In contrast to the other parameters, the oxygen consumption linked to ATP production (*J*O_2_ATP) was not affected by CRH treatment (*J*O_2_ATP: CRH 0.5 h 0.998 ± 0.151 *J*O_2_ fold of vehicle, *p* > 0.9999; CRH 2 h 0.786 ± 0.146 *J*O_2_ fold of vehicle, *p* = 0.7877; Fig. 4D), indicating that mitochondria still maintained an appreciable level of activity. These data were confirmed by a luciferase-based assay to detect ATP levels (Fig. Suppl. 3D), which did not detect any significant difference between CRH-treated neurons and control. In line with this, CRH did not increase mitochondrial degradation (mitophagy; as detected by co-immunolabeling experiments between TOMM20 and LC3A) (Fig. Suppl. 4A), suggesting that CRH-exposed mitochondria might preserve enough functionality to prevent them from degradation.

### Neuronal activity is reduced upon CRH treatment

**Fig. 5:**
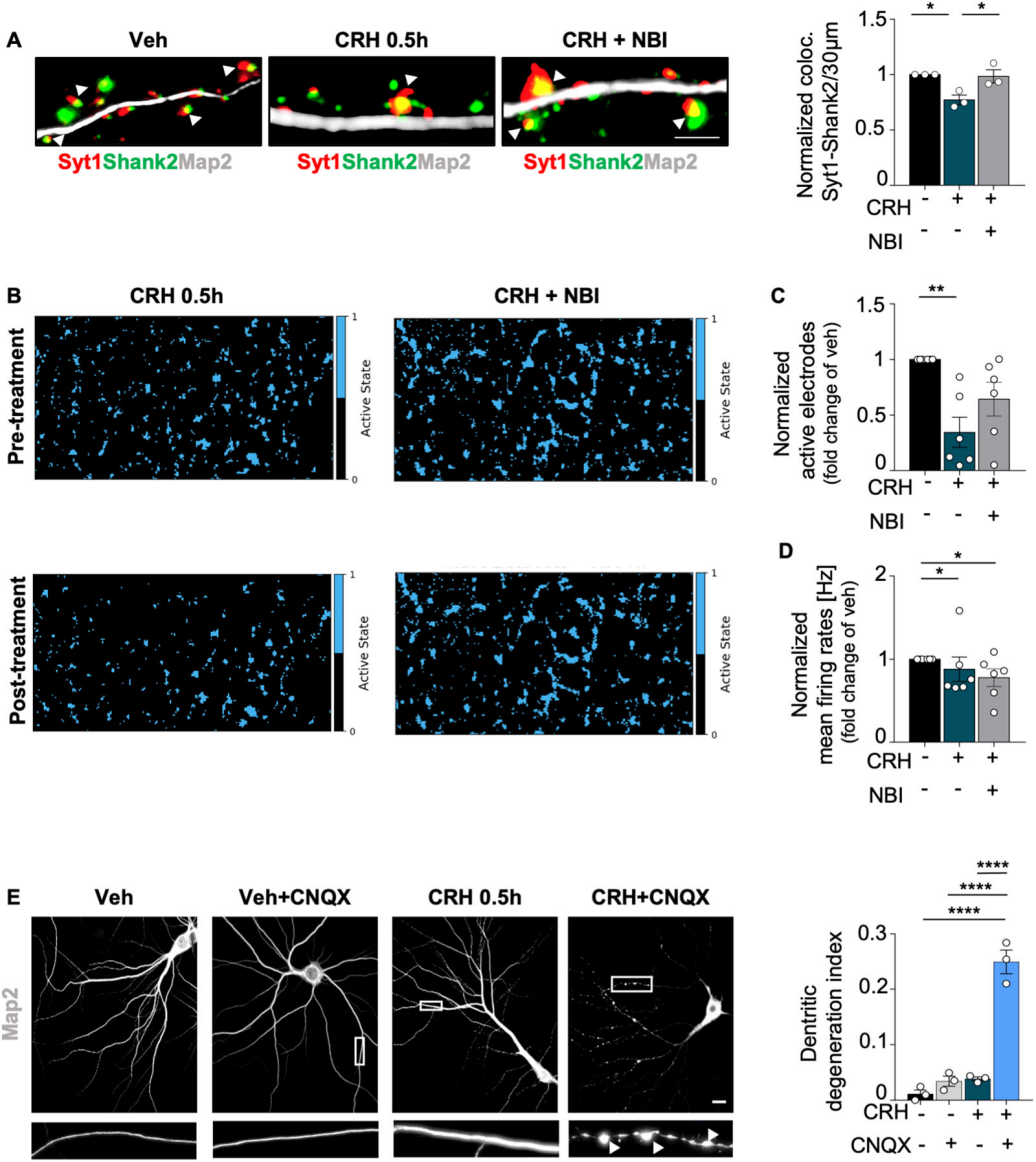
Neuronal activity is reduced upon CRH treatment. **A** IHC for MAP2 (gray), Shank2 (green) and Syt1 (red) in primary neurons treated with CRH 100 nM for 0.5 h and with CRH 0.5 h + CRHR1 Blocker NBI30775 100 nM and relative quantification of Syt1/Shank2 colocalization puncta; **B** representative images of Activity Scan Assay performed with MEA of the same cells before and after different treatments (CRH 100 nM for 0.5 h and with CRH 0.5 h + CRHR1 Blocker NBI30775 100 nM) and relative analysis of **C** active electrodes and **D** firing rates; **E** IHC for MAP2 (gray) in primary neurons after different experimental conditions using CNQX disodium salt (10 μM) (scale bar = 30 μm): vehicle, vehicle+CNQX, CRH 0.5 h, CRH 0.5 h + CNQX and relative quantification of the dendritic degeneration index. For the dendritic degeneration analysis, three different dendrites of three different neurons acquired from three different wells were analyzed for each condition in each independent experiment. All experiments were performed at DIV14. Synaptotagmin assay and dendritic degeneration experiments were performed in *N* = 3 independent replicates; MEA’s data were obtained from six wells for each treatment condition derived from two independent replicates. Data are displayed as Mean ± SEM; one-way ANOVA and Bonferroni’s post hoc comparison test were performed. Data non normally distributed were analyzed by Kruskal–Wallis non parametric test followed by Uncorrected Dunn’s multiple comparison test. (*p < 0.05, **p < 0.005, ***p < 0.0005, ****p < 0.0001).

We then investigated the effect of CRH on synaptic activity. According to our previous data^32^, in vitro feeding of anti-synaptotagmin1 (Syt1) antibody revealed that CRH induced a significant reduction of the number of active excitatory synapses (determined by colocalization between Shank2 and Syt1), which was completely prevented by co-treatment with NBI (Syt1/Shank2 colocalization: CRH 0.5 h 0.771 ± 0.043 fold of vehicle, *p* = 0.0276; 0.985 ± 0.0598 in CRH + NBI vs CRH 0.5 h, *p* = 0.0371; Fig. 5A). The reduction of synaptic contacts upon CRH correlated with a dramatically significant inhibition of neuronal activity in treated cultures: recording of the electrophysiological activity before and after treatment with a multi electrode array system (Fig. 5B) showed that CRH significantly reduced the number of active electrodes (active electrodes: CRH 0.5 h 0.3449 ± 0.1359 fold of vehicle, *p* = 0.0017), indicating a reduced number of active neurons, while CRH + NBI treatment did not induce any significant change (active electrodes: 0.6434 ± 0.151 in CRH + NBI vs CRH 0.5 h, *p* = 0.1862; Fig. 5C). Interestingly, the neuronal firing rate was reduced in CRH-treated cells, as well as in presence of the CRHR1-antagonist (firing rate: CRH 0.5 h 0.8806 ± 0.1473 fold of vehicle, *p* = 0.0474; CRH + NBI 0.7791 ± 0.1065 fold of vehicle, *p* = 0.0474; Fig. 5D).

Finally, we tested the effect of CRH in a context of inhibited synaptic activity by treating primary neurons with the stress hormone, the AMPA/kainate receptor antagonist CNQX^13,49^, or the combination of the two molecules. While CRH and CNQX-treated cells were comparable to the vehicle ones, neurons treated with both chemicals were characterized by aberrant dendritic fragmentation (dendritic degeneration index: 0.0113 ± 0.0070 in Veh vs 0.2493 ± 0.0214 in CRH + CNQX, *p* < 0.0001; 0.0342 ± 0.0090 in Veh + CNQX vs CRH + CNQX, *p* < 0.0001; 0.0385 ± 0.0035 in CRH vs CRH + CNQX, *p* < 0.0001; Fig. 5E), indicating neuronal sufferance^50^.

### CRH induces DRP1-mediated mitochondrial fission

**Fig. 6:**
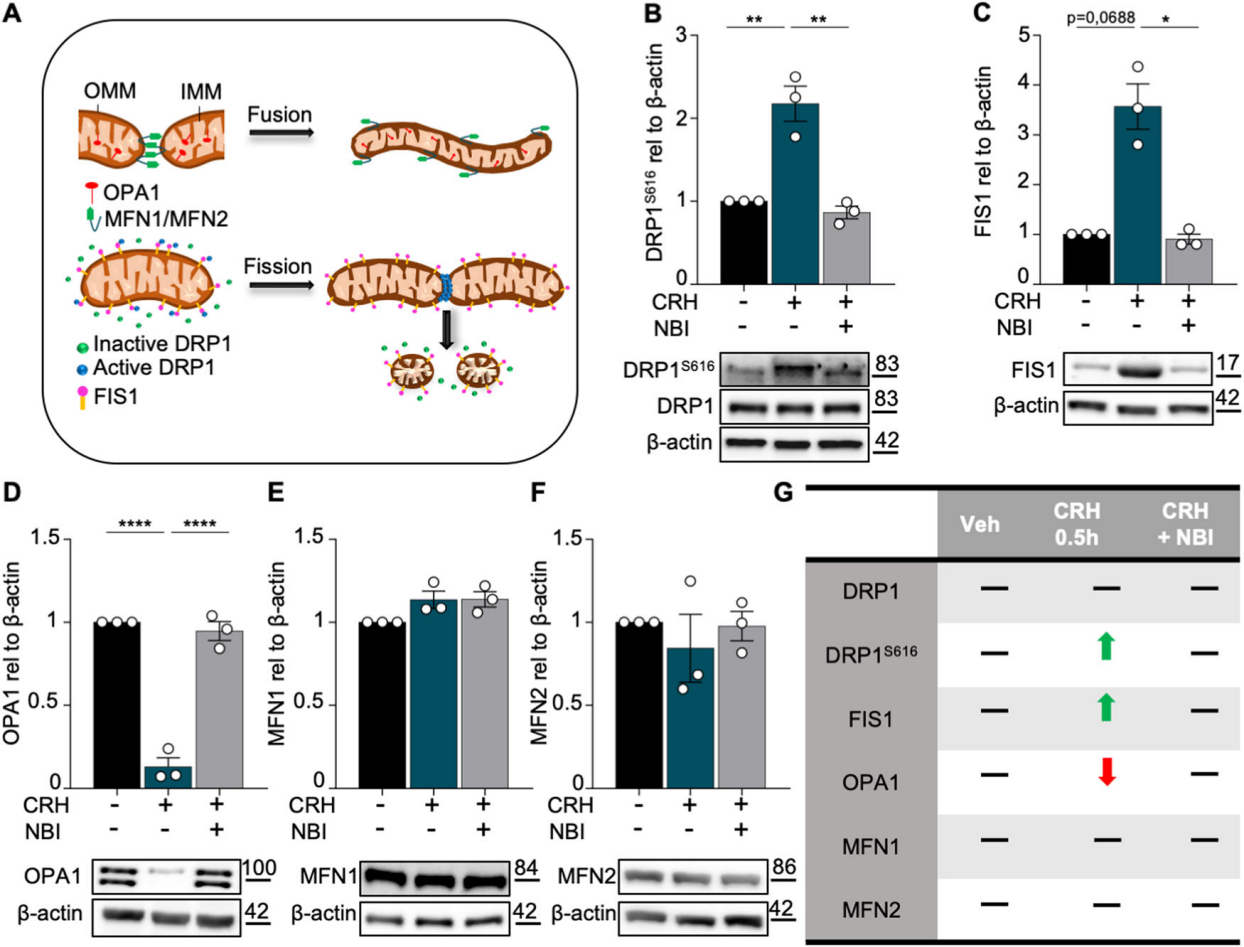
CRH induces mitochondrial fission. **A** Graphical representation of mitochondrial dynamic: fusion and fission with relative proteins (OMM = outer mitochondrial membrane; IMM = inner mitochondrial membrane). Primary hippocampal neurons at DIV14 were treated with CRH 100 nM for 0.5 h and with CRH 0.5 h + CRHR1 Blocker NBI30775 100 nM; Western Blot analysis and quantification of **B** the fission proteins DRP1^S616^, **C** FIS1 and the fusion markers **D** OPA1, **E** MFN1 and **F** MFN2. **G** Summary of the western blot results. Experiments were performed in *N* = 3 independent replicates at DIV14. Data are displayed as Mean ± SEM; one-way ANOVA and Bonferroni’s post hoc comparison test were performed; data non normally distributed were analyzed by Kruskal–Wallis non parametric test followed by Uncorrected Dunn’s multiple comparison test. (*p < 0.05, **p < 0.005, ***p < 0.0005, ****p < 0.0001).

Since the maintenance of functional mitochondria depends on a balanced fusion/fission dynamic (Fig. 6A), we then investigated the levels of several proteins involved in these processes upon CRH treatment. We found that the levels of phosphorylated (e.g. active) DRP1^S616^ and total FIS1 were significantly increased in neurons incubated with CRH (DRP1^S616^: CRH 0.5 h 2.175 ± 0.2118 fold of vehicle, *p* = 0.0021; 0.8649 ± 0.0761 in CRH + NBI vs CRH 0.5 h, *p* = 0.0012; FIS1: CRH 0.5 h 3.57 ± 0.454 fold of vehicle, *p* = 0.0688; 0.9063 ± 0.1016 in CRH + NBI vs CRH 0.5 h, *p* = 0.0229; Fig. 6B, C), indicating a strongly increased mitochondrial fission. In contrast OPA1, which is required for mitochondrial *cristae* structural organization^51^, was significantly downregulated by CRH (OPA1: CRH 0.5 h 0.1303 ± 0.0545 fold of vehicle, *p* < 0.0001; 0.9472 ± 0.0571 in CRH + NBI vs CRH 0.5 h, *p* < 0.0001; Fig. 6D), while MFN 1 (MF 1: CRH 0.5 h 1.136 ± 0.0532 fold of vehicle, *p* = 0.1709; 1.139 ± 0.0469 in CRH + NBI vs CRH 0.5 h, *p* > 0.9999; Fig. 6E) and MFN 2 (MF 2: CRH 0.5 h 0.844 ± 0.2044 fold of vehicle, *p* > 0.9999; 0.9773 ± 0.0877 in CRH + NBI vs CRH 0.5 h, *p* > 0.9999; Fig. 6F) were not altered by the treatment. Changes in the levels of fusion and fission proteins mediated by CRH are summerized in Fig. 6G.

Notably, a significant upregulation of DRP1^S616^ was also detected in the hippocampus homogenate of mice after TxT (DRP1^S616^: TxT 1.441 ± 0.1198 fold of vehicle, *p* = 0.0212; Fig. Suppl. 5A). This indicated that, despite its simplicity, our in vitro model closely recapitulates the CRH-dependent mechanisms affecting mitochondrial dynamics occurring after peripheral trauma in vivo.

### c-Abl inhibition prevents DRP1-dependent mitochondrial fission triggered by CRH

**Fig. 7:**
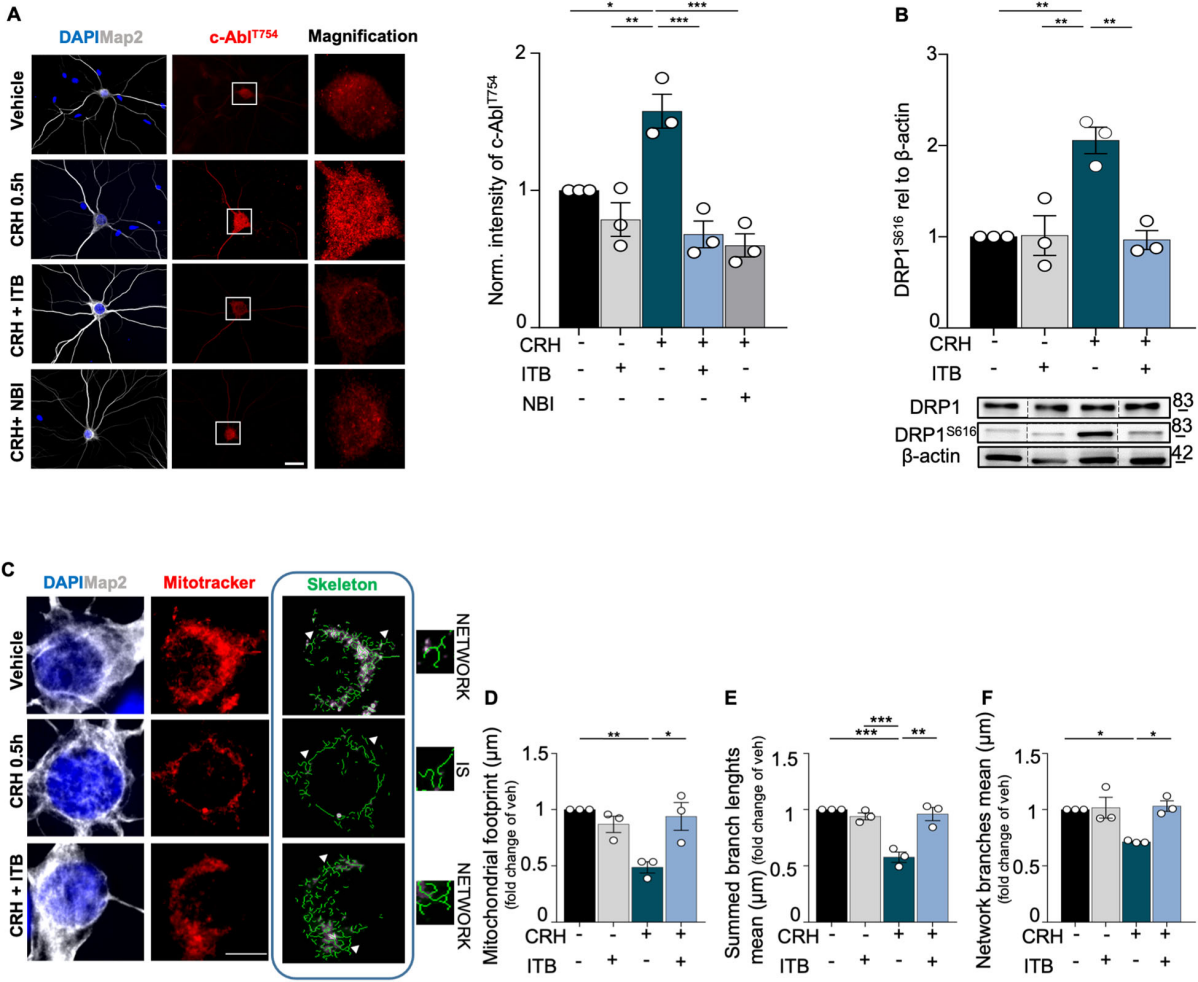
CRH-dependent c-Abl activation leads to mitochondrial fission, inducing mitochondrial network fragmentation. **A** IHC for MAP2 (gray) and c-Abl^T754^ (red) in primary hippocampal neurons after different experimental conditions using IMATINIB (ITB), a selective c-Abl blocker (scale bar = 30 μm): vehicle, vehicle + ITB 3 μM, CRH 100 nM for 0.5 h, CRH 100 nM + ITB 3 μM and CRH 100 nM + CRHR1 Blocker NBI30775 100 nM with relative c-Abl^T754^ intensity analysis. Nine different neurons acquired from three different wells were analyzed for each condition in each independent experiment. **B** WB analysis and quantification of DRP1^S616^ expression. **C** Somatic mitochondrial network complexity analysis after IHC, using the same experimental conditions (scale bar = 5 μm): MAP2 (gray), mitochondria labeled with MitoTracker^TM^ (red), mitochondrial skeletonized structures (green) (white arrows indicates examples of networks and IS) and **D**–**F** relative network parameters quantification. Somatic mitochondrial networks were analyzed in six neurons for each condition for each different independent preparation. Experiments were performed in *N* = 3 independent replicates at DIV14. Data are displayed as Mean ± SEM; one-way ANOVA and Bonferroni’s post hoc comparison test were performed; data non normally distributed were analyzed by Kruskal–Wallis non parametric test followed by Uncorrected Dunn’s multiple comparison test. (*p < 0.05, **p < 0.005, ***p < 0.0005, ****p < 0.0001).

We then aimed to identify the signaling cascades involved in the mitochondrial structural alterations and fission downstream of the CRHR1 activation. Having previously shown that CRH triggers a strong increase in the phosphorylation levels of c-Abl^32^, which is responsible for the activation of DRP1^52^, we investigated its involvement in the CRH-induced mitochondrial phenotype. First, we confirmed the increased phosphorylation of c-Abl^T754^ in CRH-treated hippocampal neurons (intensity of c-Abl^T754^: CRH 0.5 h 1.578 ± 0.1231 fold of vehicle, *p* = 0.0178; Fig. 7A). In addition, we found that this effect was completely prevented by the selective c-Abl inhibitor Imatinib (ITB), and by the CRHR1 antagonist NBI as well, indicating that CRHR1 activation triggers c-Abl phosphorylation (intensity of c-Abl^T754^: 0.6806 ± 0.0973 in CRH + ITB vs CRH 0.5 h, *p* = 0.0006; 0.6 ± 0.0849 in CRH + NBI vs CRH 0.5 h, *p* = 0.0003; 0.7877 ± 0.1227 in Veh + ITB vs CRH 0.5 h, *p* = 0.0018; Fig. 7A). Interestingly, ITB also completely inhibited the CRH effect on DRP1 phosphorylation in co-treated cultures, but had no effects when applied alone in resting conditions (DRP1^S616^: CRH 0.5 h 2.056 ± 0.1458 fold of vehicle, *p* = 0.0043; CRH 0.5 h vs 1.014 ± 0.2174 in Veh + ITB, *p* = 0.0047 and vs 0.9661 ± 0.1037 in CRH + ITB, *p* = 0.0035; Veh+ITB 1.014 ± 0.2174 fold of vehicle, *p* = 0.9999; Fig. 7B). Moreover, by investigating the mitochondrial network with MitoTracker™ in immunostaining (Fig. 7C), we observed that inhibition of c-Abl with ITB successfully rescued the alterations in mitochondrial footprint (mitochondrial footprint: CRH 0.5 h 0.4867 ± 0.0495 µm^2^ fold of vehicle, *p* = 0.0062; 0.9395 ± 0.1237 µm^2^ in CRH + ITB vs CRH 0.5 h, *p* = 0.0401; Fig. 7D) and network branching (summed branch length mean: CRH 0.5 h 0.5771 ± 0.0462 μm fold of vehicle, *p* = 0.0005; 0.94 ± 0.0304 μm in Veh + ITB vs CRH 0.5 h, *p* = 0.0013; 0.9602 ± 0.05872 μm in CRH + ITB vs CRH 0.5 h, *p* = 0.0009; Fig. 7E; network branches mean: CRH 0.5 h 0.7136 ± 0.0063 μm fold of vehicle, *p* = 0.0401; 1.031 ± 0.0464 μm in CRH + ITB vs CRH 0.5 h, *p* = 0.0166; Fig. 7F) induced by the CRH treatment. Thus, c-Abl activation is selectively required for DRP1-dependent mitochondrial fission in response to CRH treatment in hippocampal neurons.l

### CRH-dependent mitochondrial fission requires NF-kB activity and DRP1 nitrosylation

**Fig. 8:**
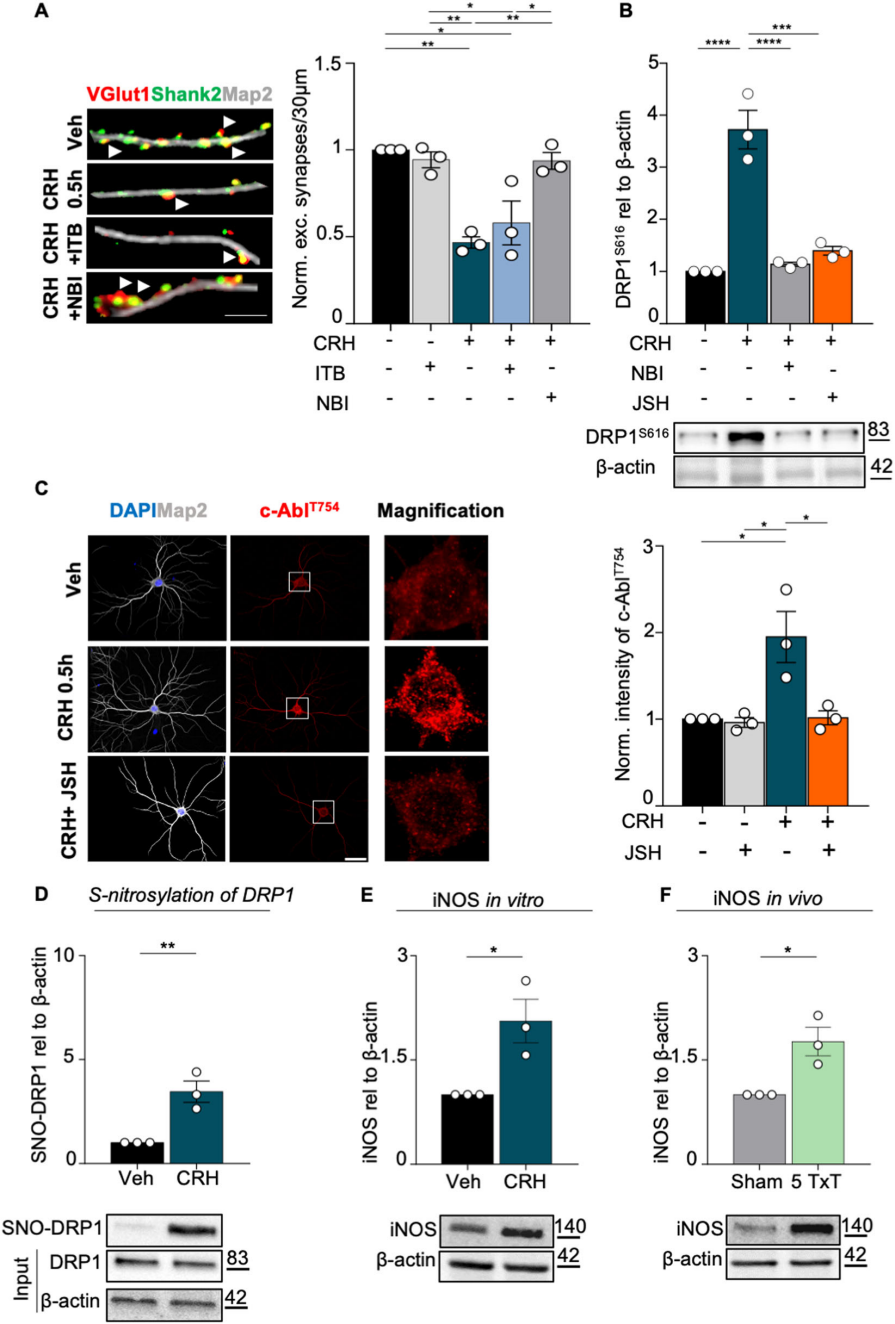
CRH-c-Abl dependent mitochondrial fission requires NF-kB activity. **A** IHC for MAP2 (gray), VGlut1 (red) Shank2 (green) after different treatments (scale bar = 5 μm). White arrowheads indicate the co-localization between VGlut1 and Shank2. Quantification of excitatory synapses number (co-localization of Shank2/Vglut1/30μm of dendrites). Three different dendrites of three different neurons acquired from three different wells were analyzed for each condition in each independent experiment. **B** WB analysis of DRP1^S616^ expression, blocking the NF-κB pathway with the nuclear translocation blocker JSH-23. **C** IHC for MAP2 (gray) and c-Abl^T754^ (red) in primary neurons after different experimental conditions using JSH-23 (scale bar = 30 μm): vehicle, vehicle + JSH 10 μM, CRH 100 nM for 0.5 h, CRH 100 nM + JSH 10 μM with relative c-Abl^T754^ intensity analysis. Nine different neurons acquired from three different wells were analyzed for each condition in each independent experiment. Representative western blot and relative quantification of: **D** S-nitrosylation levels of DRP1 and **E** of iNOS expression following CRH treatment; **F** iNOS expression levels in 5 Txt mice. Experiments were performed in *N* = 3 independent replicates at DIV14. Data are displayed as Mean ± SEM; one-way ANOVA and Bonferroni’s post hoc comparison test were performed (*p < 0.05, **p < 0.005, ***p < 0.0005, ****p < 0.0001).

Considering that CRH treatment significantly reduces neuronal activity and the number of excitatory synapses, we tested whether inhibition of c-Abl might also rescue this neuronal alteration, besides preventing mitochondrial fission. In agreement with the reduction of neuronal activity (Fig. 5B–D), CRH induced a significant loss of VGlut1-Shank2 positive excitatory synapses in primary neurons, while co-application of NBI protected them from synaptic degradation^32^. To our surprise, ITB failed in rescuing the synaptic loss induced by CRH (number of excitatory synapses: CRH 0.5 h 0.467 ± 0.0328 fold of vehicle, I = 0.0018; CRH + ITB 0.9365 ± 0.0481 fold of vehicle, *p* = 0.0106; 0.9427 ± 0.0457 in VEH + ITB vs CRH 0.5 h, *p* = 0.0044 and vs CRH + ITB, *p* = 0.0284; 0.9365 ± 0.0481 in CRH + NBI vs CRH 0.5 h, *p* = 0.0048 and vs CRH + ITB, *p* = 0.0316; Fig. 8A), indicating that the effect of CRHR1 activation might be mediated by other effectors acting upstream of c-Abl.

To better dissect these molecular mechanisms, we tested whether inhibition of NF-kB^53,54^ by the specific JSH-23 inhibitor (which rescues the loss of synapses induced by CRH^32^; might prevent the mitochondrial alterations triggered by CRH. We found that JSH-23 inhibited the activation of DRP1 (when co-applied with CRH) in a way that was comparable to NBI (DRP1^S616^: CRH 0.5 h 3.724 ± 0.3693 fold of vehicle, *p* < 0.0001; CRH 0.5 h vs 1.139 ± 0.0369 in CRH + NBI, *p* < 0.0001 and vs 1.398 ± 0.0832 in CRH + JSH, *p* = 0.0001; Fig. 8B). Moreover, JSH also completely abolished the activation of c-Abl triggered by CRHR1 activation (intensity of c-Abl^T754^: CRH 0.5 h 1.948 ± 0.2953 fold of vehicle, *p* = 0.0155; CRH 0.5 h vs 0.9623 ± 0.0572 in Veh + JSH, *p* = 0.0123 and vs 1.016 ± 0.0793 in CRH + JSH, *p* = 0.0171; Fig. 8C), indicating that CRH-induced mitochondrial fission depends on the activation of NF-kB pathway. We then tested whether increased levels of nitric oxide (NO), whose regulation has been shown to be also CRH dependent^55^ might represent the trigger activating the signaling cascade leading to mitochondrial alterations in our model. We found that CRH increased the levels of nitrosylated DRP1 (SNO-DRP1: CRH 0.5 h 3.459 ± 0.5115 fold of vehicle, *p* = 0.0086; Fig. 8D), as well as the levels of the inducible NO synthase (iNOS), (iNOS: CRH 0.5 h 2.061 ± 0.312 fold of vehicle, *p* = 0.0272; Fig. 8E). Interestingly, CRHR1 activation significantly decreased the levels of the proinflammatory cytokines IL-6 and IL-17 (IL-6: CRH 0.5 h 0.4875 ± 0.0082 fold of vehicle, *p* < 0.0001; IL-17: CRH 0.5 h 0.7036 ± 0.0503 fold of vehicle, *p* = 0.0287; Fig. Suppl. 6A, B) in cultured neurons, suggesting that both mitochondrial and synaptic alterations triggered by CRH depend on Nf-KB activation through increased NO levels. Notably, we observed similar effects in our in vivo trauma model: infact, TxT animals showed increased levels of hippocampal iNOS (iNOS: 5 TxT 1.766 ± 0.2044 fold of Sham, *p* = 0.0200; Fig. 8F), together with a significant reduction of IL-6 and IL-7 when compared to Sham ones (IL-6: 5 TxT 0.5052 ± 0.0652 fold of vehicle, *p* = 0.0016; IL-17: 5 TxT 0.2102 ± 0.0317 fold of vehicle, *p* < 0.0001; Fig. Suppl. 6C–D). All in all, our results indicate NO as a specific mediator of the alterations occurring when hippocampal neurons are exposed, in vitro as well as in vivo, to CRH.

## Discussion

Mitochondrial alterations are a pathological feature shared by several synaptopathies contributing to neuronal sufferance, synapse loss, and eventually neuronal death. In Alzheimer’s disease, the mitochondrial accumulation of amyloid-β impaires the functionality of these organelles, sustaining disease progression^56^, while altered activity of the mitochondrial complex I has been observed in Parkinson’s disease^57^. Likewise, cortical neurons deprived of glucose and oxygen display increased fission linked to reduced OPA1 levels, which eventually leads to neuronal death^58^; notably, mitochondrial activation of caspase-3 signaling not only triggers neuronal apoptosis, but also controls neuronal plasticity^59^. Thus, mitochondrial alterations, synapse loss, and neuronal death appear to be typical and correlated detrimental events in neurodegenerative processes. In this scenario, the CRH-mediated loss of hippocampal synapses described both in vivo and in vitro, appears as an exception. In fact, on one side the synaptic and cognitive phenotypes induced by TxT (mediated by CRH) completely recover after 18 days^32^ without any pharmacological intervention. On the other side, after long-term application of CRH (5 h), cultured neurons spontaneously recover and the respiratory chain machinery re-gains physiological activity levels. Accordingly, CRH has a half life of ~30 min in humans^60^. Therefore, this in vitro model, although presenting important metabolic differences if compared to the brain (as summarized in Dienel^61^), might represent a *bona fide* model resembling a single burst of CRH triggered by a stressful stimulus (such as trauma). CRH acts on neuronal CRHR1 and thereby activates several intracellular pathways that induce metabolic, as well as structural alterations. Those alterations seem to secure neuronal survival and stability of essential circuits in order to overcome a harmful period. In fact, CRH induces a general reduction of synaptic activity in cultured neurons, which might reasonably occur also in the in vivo trauma model. Here, the loss of synaptic contacts upon CRH exposure triggered by TxT correlates with a general worsening of intellectual performances persisting until hippocampal synapses are restored^32^. Furthermore, the mitochondrial alterations observed in vitro closely resemble those detected in TxT animals, supporting the translational relevance of the results obtained with primary neurons. In fact, the trauma-induced synaptic loss does not depend on increased neuronal apoptosis and in the present study we detected signs of neuronal sufferance only when CRH was co-administered with CNQX. This might explain why, despite undergoing drastic morphological rearrangements, CRH-treated mitochondria still produce ATP levels comparable to untreated neurons. In light of these findings, it is reasonable to speculate that neurons still require a considerable amount of energy to maintain viability upon CRH treatment. In fact, those synapses not undergoing autophagic degradation upon CRHR1 activation maintain their functionality^32^, highlighting the importance of maintaining a certain degree of neuronal activity to avoid neuronal death and allow efficient recovery after an insult^62^. This indicates that the exposure to CRH triggers dynamic synaptic modifications similar to those of long-term depression (LTD), in which preserved synapses keep enough activity aimed to the maintenance of the neuronal population for a full recovery after the triggering signal. In fact, neuronal activity has been shown to exert a neuroprotective effect in several neurological conditions, while inhibition of neuronal firing increases neuronal stress and apoptosis^63,64^.

Given the well-described functional relation between synapses and mitochondria, our results raised the question whether mitochondrial alterations and loss of excitatory synapses are independent (but still convergent) events. Since CRHR1 is located at the synapse and CRH-dependent synaptic autophagic degradation requires NF-kB activation^32^, we speculated that mitochondrial dysfunctions might occur in response of these first event. In fact, inhibition of NF-kB nuclear translocation prevents both synaptic degradation and mitochondrial fission induced by CRH, without triggering an overall pro-inflammatory signaling cascade. Interstingly, the levels of IL-6 and IL-17 (which have been linked to increased neuronal death^65–67^ were significantly downregulated in CRH-treated neurons, and in TxT mice as well. In contrast CRH increased (in vivo and in vitro) the levels of iNOS, together with those of nitrosylated DRP1, suggesting a specific role played by nitric oxide upon CRHR1 activation. Previous studies have shown that the NOS inhibitor L-NAME reduces the CRH-mediated ACTH release^68^. Thus, although Nf-KB nucler function may be required for the activation, among others, of synaptic autophagy, its involvement in mitochondrial fission seems to depend on other mechanisms than its canonical activation. Bottero and collaborators^69^ have detected NF-kB in purified mitochondrial fraction, while a later study located its subunits p50 and p65 to the inner matrix of these organelles^70^. Moreover, it has been recently shown that TNF-alpha treatment induces OPA1-mediated mitochondrial fusion through NF-kB^71^, and that NF-kB controls the expression of COX III, which is a subunit of the complex IV of the respiratory chain^70^. Moreover, CRH-induced mitochondrial fission can be rescued also by inhibiting c-Abl activity, indicating a complex molecular cascade set in motion by CRH exposure. In this context, the increased activity of iNOS and the recruitment of Nf-KB, together with the upregulation of DRP1-dependent mitochondrial fission, might be part of the adaptive response set in motion by resilient neurons in order to survive the external insult triggering CRH release (such as TxT). A similar beneficial role has been described in cardiomyocytes, which are protected from excessive oxidative stress by physiological levels of S-nitrosylation^72^. In contrast, exacerbated S-nitrosylation induces synaptic aberrations similar to those observed by our group^73^. This suggests that repeated and prolonged bursts of CRH might eventually lead to irreversible neuronal damages: in fact, patients suffering from multiple severe traumas display a worse neurologic recovery (and higher mortality rate) than those undergoing only isolated injuries^74^.

All in all, our study elucidates the mechanism by which neurons rearrange their mitochondrial network in response to CRH-mediated synaptic loss. We show also that the alterations hereby dissected in cultured neurons resemble those occurring in vivo upon trauma, and can be prevented by pharmacological interventions at different steps of the signaling cascade activated by the stress hormone, conferring therapeutic relevance to our results. Mitochondrial and NF-kB alterations have been linked in an Alzheimer’s disease model, while c-Abl inhibition has been recently shown to be neuroprotective in ALS^75^. Furthermore, aberrant nitrosylation of key proteins involved in metabolic processes, including DRP1, is thought to be a driving pathomechanism in neurodegenerative pathologies^76^, such as Hungtington´s disease^77^.

**Fig. 9:**
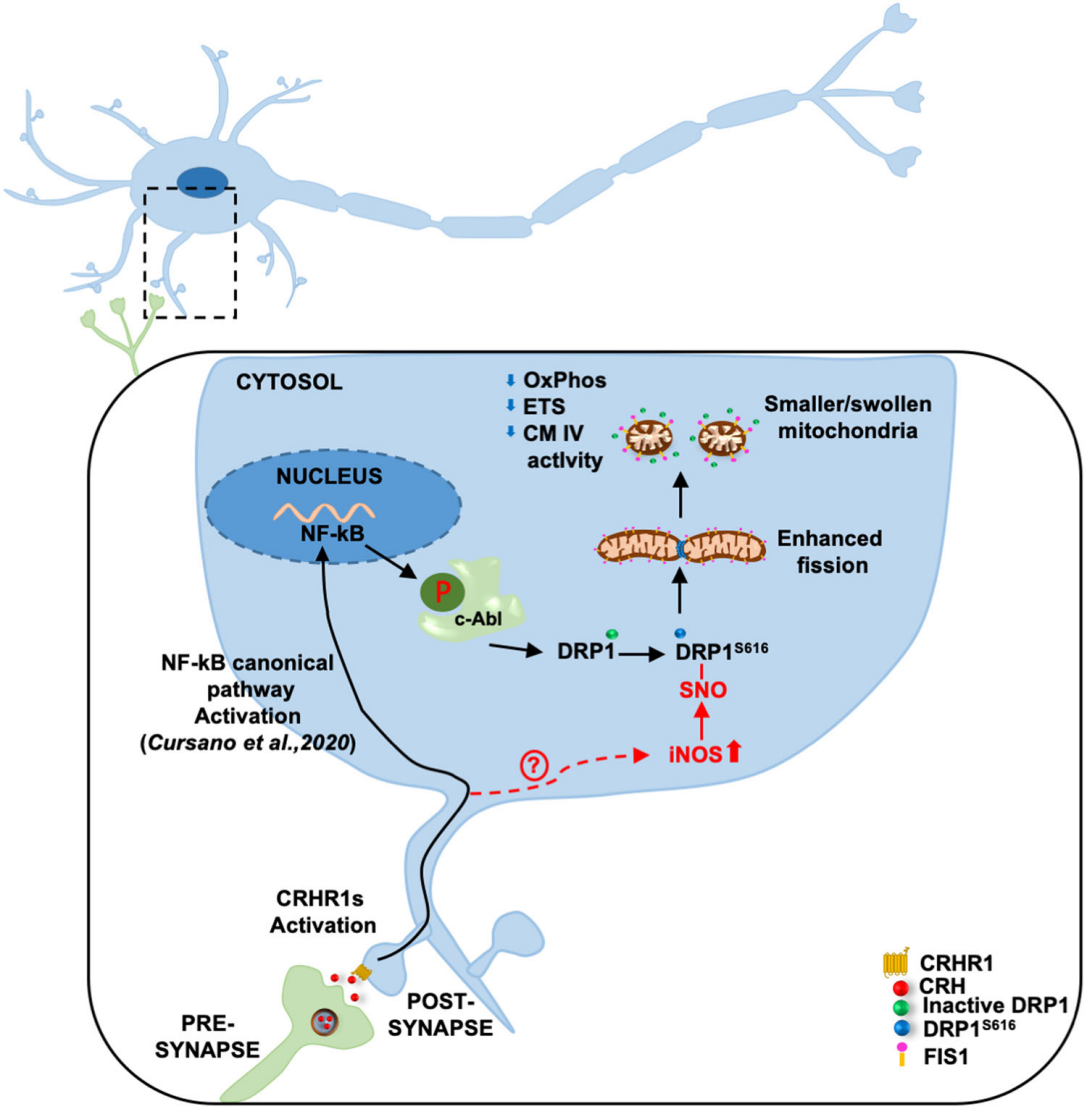
Schematic representation of the proposed molecular mechanism. CRHR1 activation triggers a signaling cascade leading to the activation of Nf-KB and increased iNOS activity. This eventually leads to increased phospshporylation and nytrosilation of DRP1, which drives the morphological and bioenergetics alterations of mitochondria upon CRH exposure.

Thus, this work broadens the spectrum of neurological conditions, characterized by synaptic alterations, mitochondrial abnormalities, and oxidative stress, that might benefit from modulation of the intracellular signaling cascades involving nitric oxide, Nf-KB, and DRP1 triggered by CRH (Fig. 9).

## Supplementary information

Supplementary methods

Figure Supplement 1

Figure Supplement 2

Figure Supplement 3

Figure Supplement 4

Figure Supplement 5

Figure Supplement 6

Supplementary Figure Legends
