# Supplementary material for: Corticotropin-releasing hormone (CRH) alters mitochondrial morphology and function by activating the NF-kB-DRP1 axis in hippocampal neurons
Source: Cell Death Dis. 2020 Nov 23;11(11):1004. doi: 10.1038/s41419-020-03204-3 (PMC7683554; doi:10.1038/s41419-020-03204-3)
Supplement: Supplementary file 1 — Supplementary methods [file 41419_2020_3204_MOESM1_ESM.docx]

**Corticotropin Releasing Hormone (CRH) alters mitochondrial morphology and function by activating the NF-kB-DRP1 axis in hippocampal neurons**

Chiara R Battaglia^1,2^, Silvia Cursano^1,2^, Enrico Calzia^3^, Alberto Catanese^1^, Tobias M Boeckers ^1,4^

Affiliation: ^1^ Institute of Anatomy and Cell Biology, Ulm University, Ulm (Germany)

^2^ International Graduate School, Ulm University, Ulm (Germany)

^3^ Institute for Anesthesiologic Pathophysiology and Process Engineering, Ulm University, Ulm, Germany

^4^ DZNE, Ulm site, Ulm, Germany

Corresponding Authors: Prof. Dr. Tobias Böckers, Dr. Alberto Catanese,

Institute of Anatomy and Cell Biology,

Ulm University, Albert-Einstein-Allee 11, 89081 Ulm DE

email: [tobias.boeckers@uni-ulm.de](mailto:tobias.boeckers@uni-ulm.de), [alberto.catanese@uni-ulm.de](mailto:alberto.catanese@uni-ulm.de)

**Supplementary Materials and Methods**

***ATP levels measurement by luciferase-based Cell Viability Assay (CellTiter-Glo^Ⓡ^ Assay, Promega)***

CellTiter-Glo^®^ (Promega, #G7570) Luminescent Cell Viability Assay kit was run according to the manual. Primary hippocampal neurons were plated on 24-well plate and exposed to CRH (100 nM) for 0.5h and 2 hours or vehicle (DMSO). After the treatment, 500 μl of CellTiter-Glo^®^ Reagents (previously thawed and equilibrated at room temperature) were added in each well and mixed for 2 min on an orbital shaker to induce cell lysis. Then, the plates were kept at room temperature for 10 min to stabilize luminescence signal and, finally, the emitted light was quantified by the luminescence reader Cytation™3 Imaging Multi-Mode Reader (BioTek^®^) using Gen5™ software, whereby the measured luminescence signal is directly proportional to the amount of ATP present in each sample. Experiments were performed in N=4 independent replicates. One-way ANOVA and Bonferroni’s *post hoc* comparison test were performed. Data were analyzed with GraphPad Prism (Version 7.0) and displayed as Mean ± SEM.
