# Supplementary figures and images for: Corticotropin-releasing hormone (CRH) alters mitochondrial morphology and function by activating the NF-kB-DRP1 axis in hippocampal neurons
Source: Cell Death Dis. 2020 Nov 23;11(11):1004. doi: 10.1038/s41419-020-03204-3 (PMC7683554; doi:10.1038/s41419-020-03204-3)

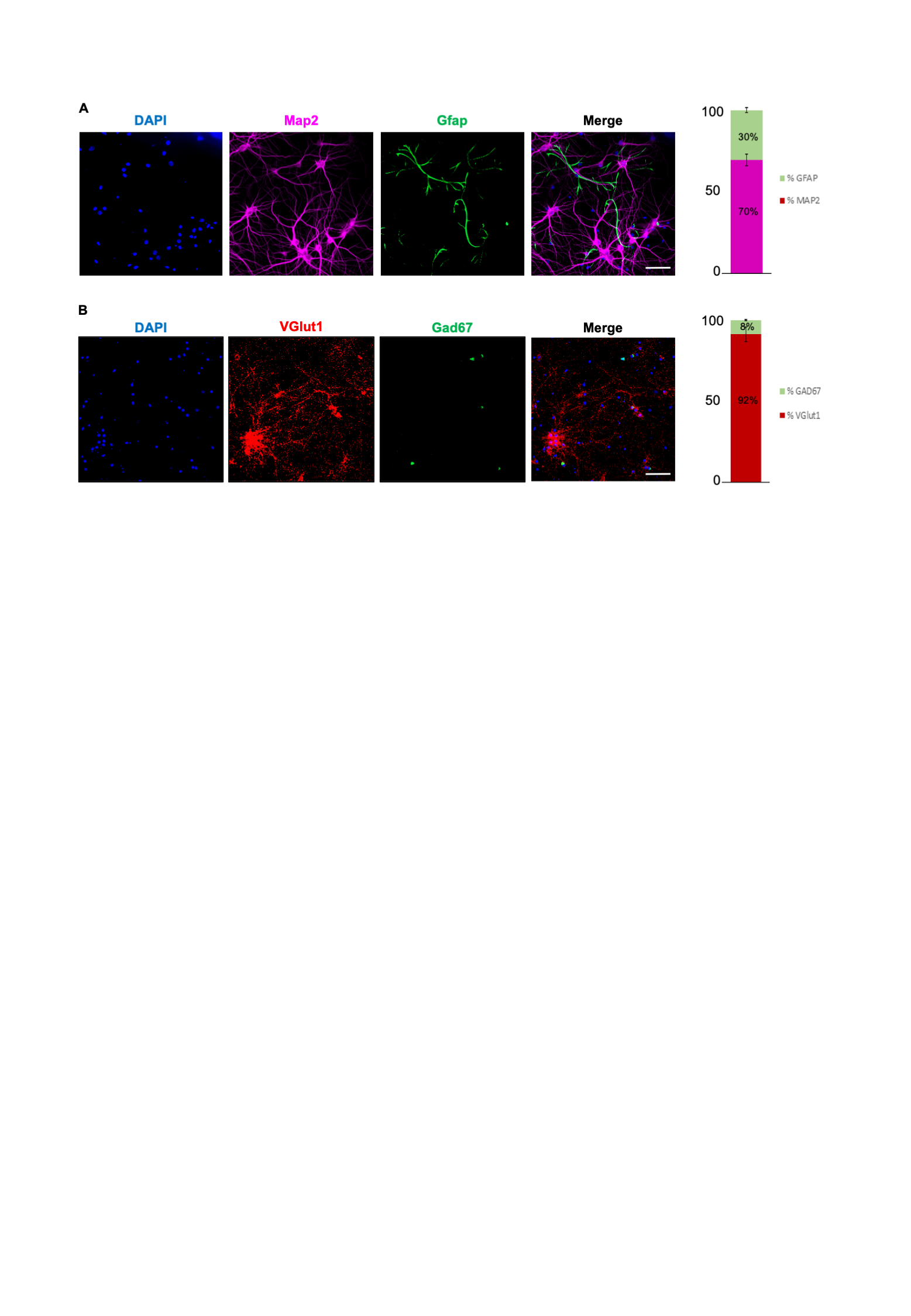

Supplement: Supplementary file 2 — Figure Supplement 1 [file 41419_2020_3204_MOESM2_ESM.tif]

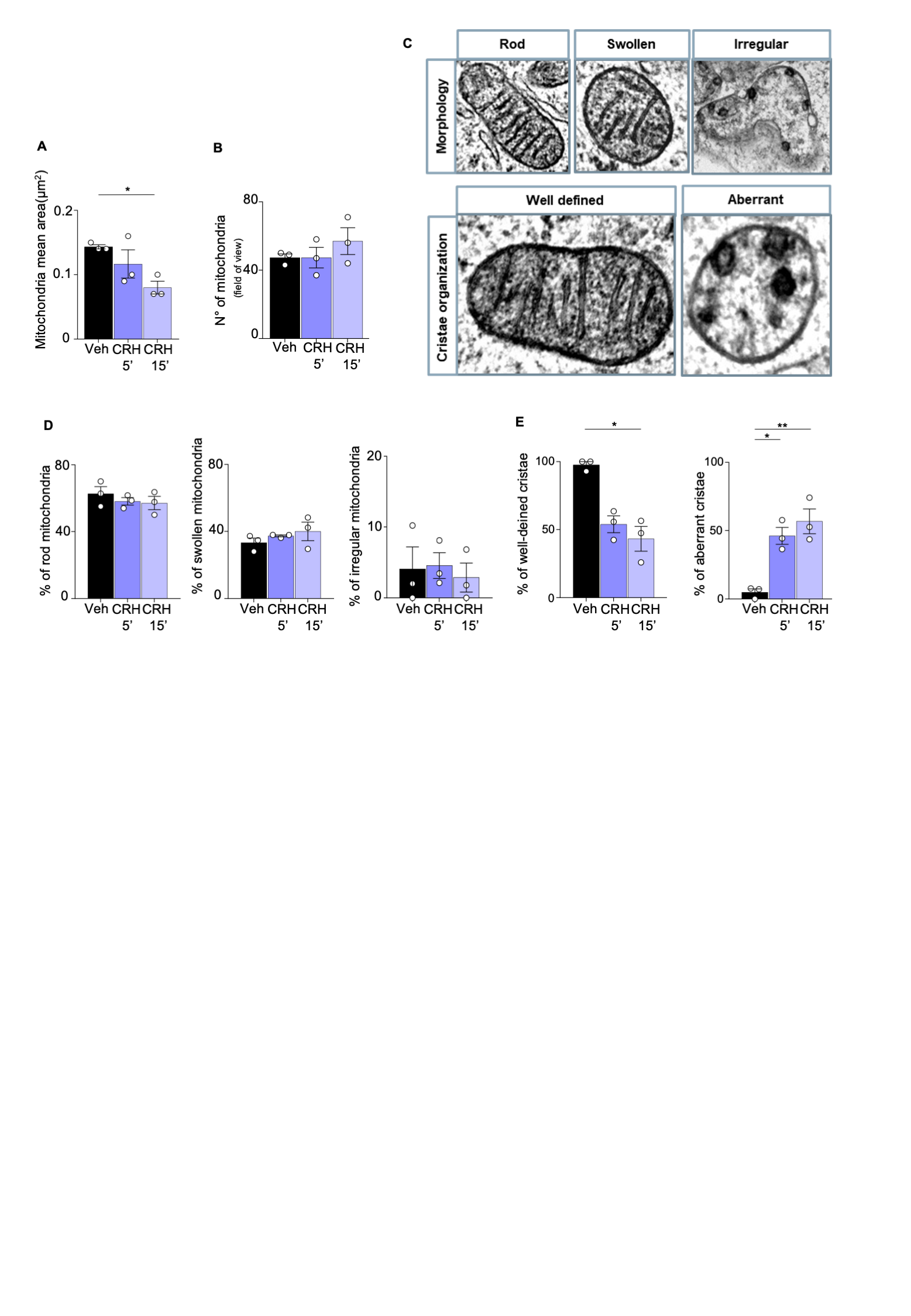

Supplement: Supplementary file 3 — Figure Supplement 2 [file 41419_2020_3204_MOESM3_ESM.tif]

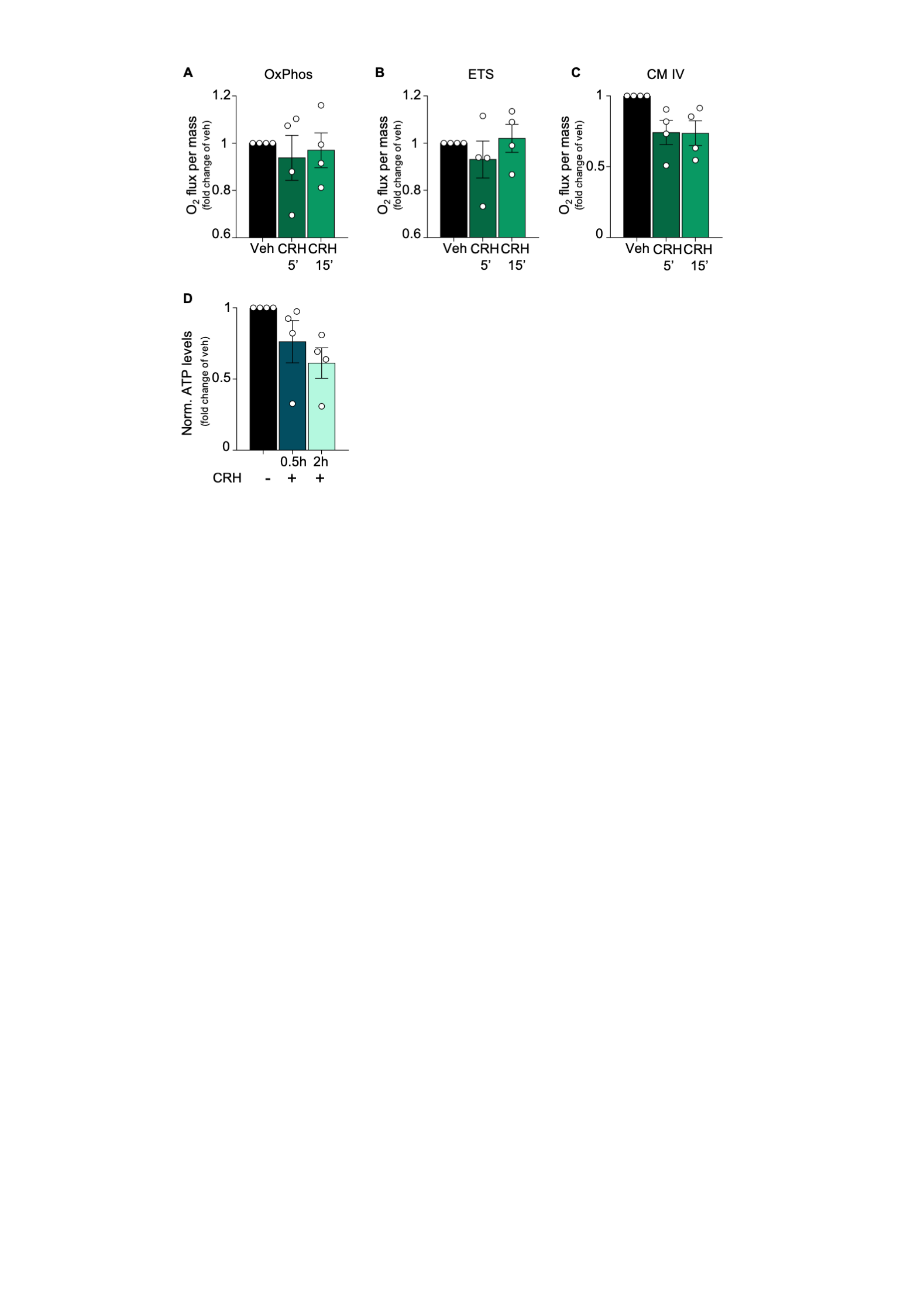

Supplement: Supplementary file 4 — Figure Supplement 3 [file 41419_2020_3204_MOESM4_ESM.tif]

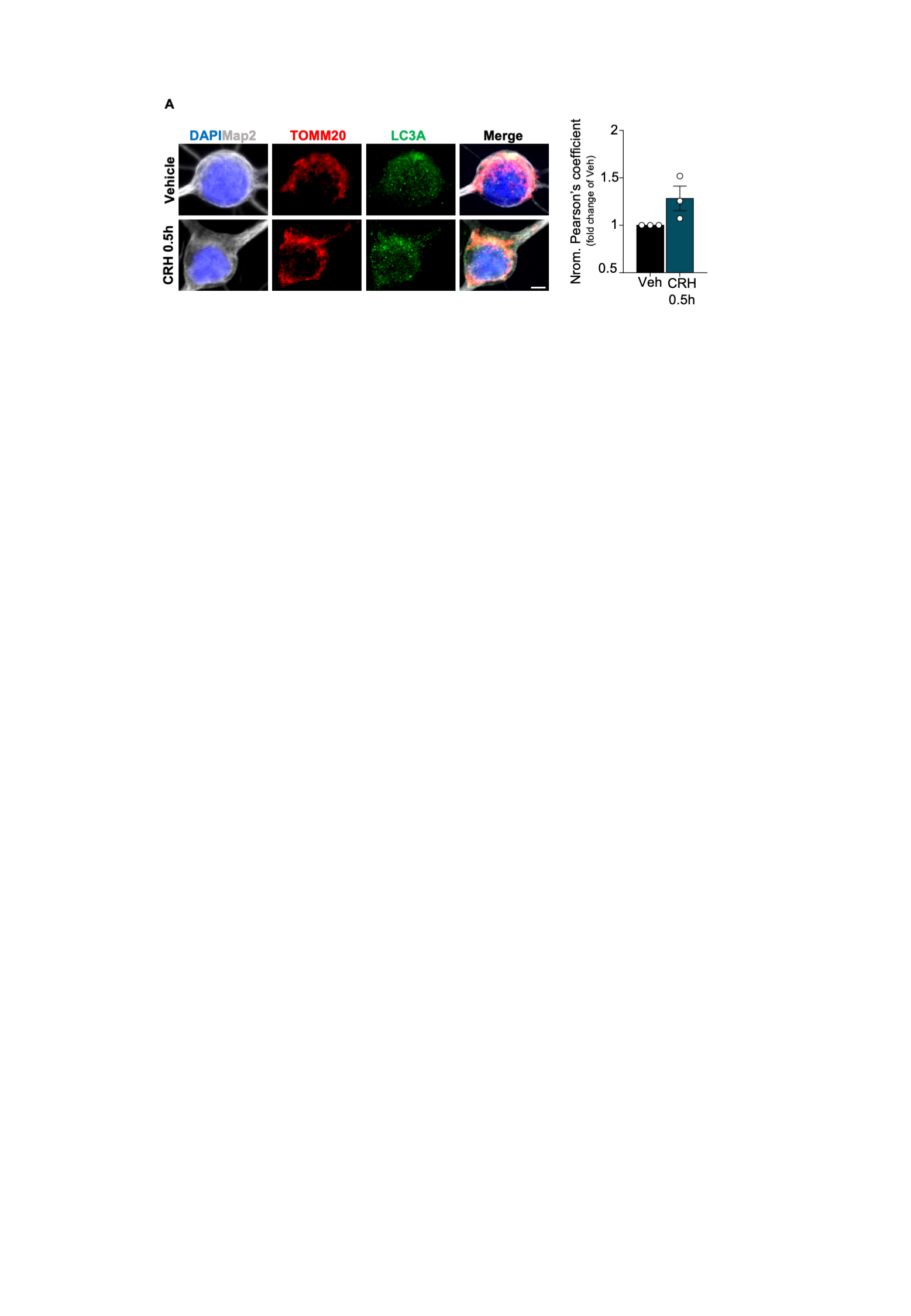

Supplement: Supplementary file 5 — Figure Supplement 4 [file 41419_2020_3204_MOESM5_ESM.tif]

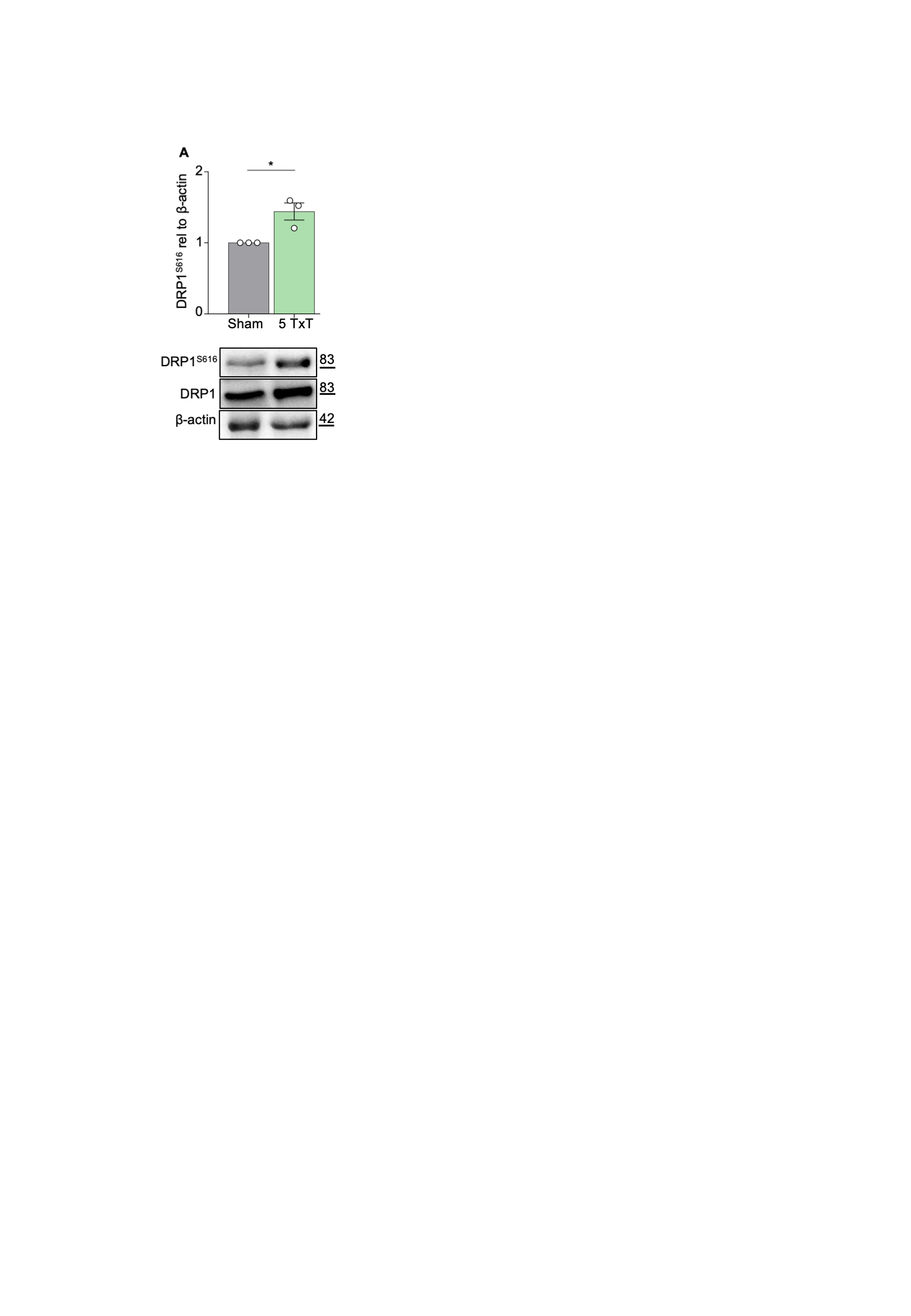

Supplement: Supplementary file 6 — Figure Supplement 5 [file 41419_2020_3204_MOESM6_ESM.tif]

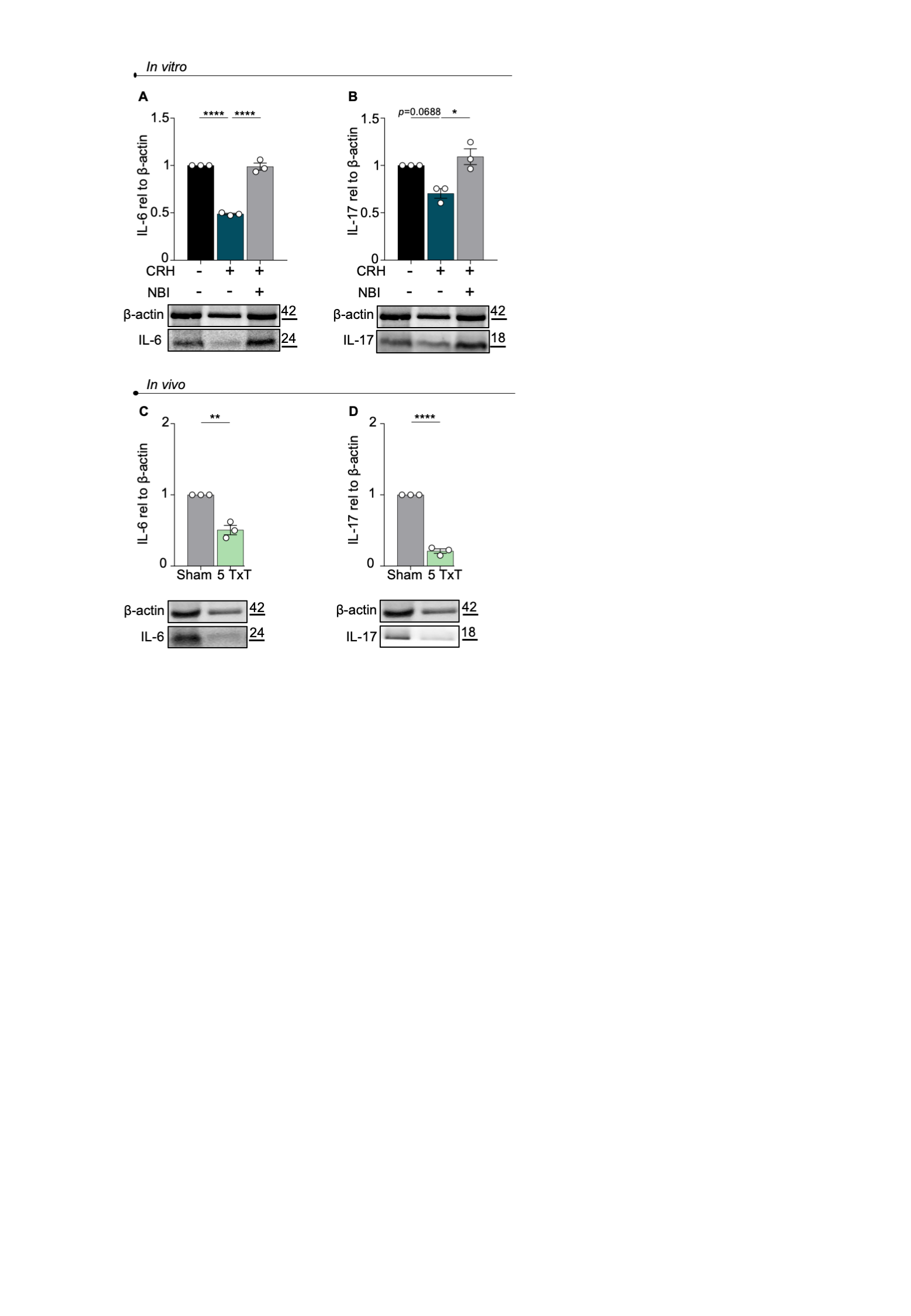

Supplement: Supplementary file 7 — Figure Supplement 6 [file 41419_2020_3204_MOESM7_ESM.tif]
