# Supplementary material for: Corticotropin-releasing hormone (CRH) alters mitochondrial morphology and function by activating the NF-kB-DRP1 axis in hippocampal neurons
Source: Cell Death Dis. 2020 Nov 23;11(11):1004. doi: 10.1038/s41419-020-03204-3 (PMC7683554; doi:10.1038/s41419-020-03204-3)
Supplement: Supplementary file 8 — Supplementary Figure Legends [file 41419_2020_3204_MOESM8_ESM.docx]

**Corticotropin Releasing Hormone (CRH) alters mitochondrial morphology and function by activating the NF-kB-DRP1 axis in hippocampal neurons**

Chiara R Battaglia^1,2^, Silvia Cursano^1,2^, Enrico Calzia^3^, Alberto Catanese^1^, Tobias M Boeckers ^1,4^

Affiliation: ^1^ Institute of Anatomy and Cell Biology, Ulm University, Ulm (Germany)

^2^ International Graduate School, Ulm University, Ulm (Germany)

^3^ Institute for Anesthesiologic Pathophysiology and Process Engineering, Ulm University, Ulm, Germany

^4^ DZNE, Ulm site, Ulm, Germany

Corresponding Authors: Prof. Dr. Tobias Böckers, Dr. Alberto Catanese,

Institute of Anatomy and Cell Biology,

Ulm University, Albert-Einstein-Allee 11, 89081 Ulm DE

email: [tobias.boeckers@uni-ulm.de](mailto:tobias.boeckers@uni-ulm.de), [alberto.catanese@uni-ulm.de](mailto:alberto.catanese@uni-ulm.de)

**Supplementary Figure legends**

**Supplementary Figure S1.** Cell culture characterization. **A** IHC for MAP2 (magenta) and GFAP (green) to detect the percentage of neuronal and glial cells in the cell culture. **B** IHC for VGLUT1 (red) and GAD67 (green) in primary neurons (scale bar = 30 μm) to detect the percentage of excitatory and inhibitory neurons. Experiments were performed in N=3 independent replicates at DIV14. Data are displayed as Mean ± SEM; one-way ANOVA and Bonferroni’s post hoc comparison test were performed (*p<0.05, **p<0.005, ***p<0.0005, ****p<0.0001).

**Supplementary Figure S2.** Analysis of Electron Microscopy (EM) images of somatic hippocampal mitochondria at earlier time points of CRH incubation (Vehicle, CRH 100nM 5 min and 15 min). **A** Quantification of mitochondrial mean area/12.77μm^2^ (μm^2^) and **B** number of mitochondria. **C** Representative TEM images of mitochondrial classification regards morphology (rod, swollen, irregular) and *cristae* organization (well defined, aberrant). **D** Percentage of rod, swollen and irregular mitochondria. **E** Percentage of well-defined *cristae* and aberrant *cristae*. Experiments were performed in N=3 independent replicates at DIV14. All the mitochondria per field of view were considered. Data are displayed as Mean ± SEM; one-way ANOVA and Bonferroni’s post hoc comparison test were performed; data non normally distributed were analyzed by one-way ANOVA non parametric test Kruskal-Wallis test followed by Uncorrected Dunn's multiple comparison test. (*p<0.05, **p<0.005, ***p<0.0005, ****p<0.0001).

**Supplementary Figure S3.** Mitochondrial respiratory activity of primary neurons at earlier time points of CRH incubation (Vehicle, CRH 100nM 5 min and 15 min) measured by high-resolution respirometry using the Oxygraph-2k(R) system (OROBOROS Instruments Corp., Innsbruck, Austria). **A** Maximum OxPhos capacity, **B** maximum ETS capacity and **C** respiratory activity linked to complex IV. **D** ATP levels detected by luciferase-based assay (CellTiter-Glo^Ⓡ^ Assay, Promega) in neurons exposed to CRH for 0,5h and 2h or vehicle (DMSO). Mitochondrial respiration was quantified in terms of oxygen flux (*J*O_2_) based on the rate of change of the O_2_ concentration in the chambers after normalization to the total cell number: pmol O_2_/(s*10^6^ cells). Experiments were performed in N=4 independent replicates at DIV14. Data are displayed as Mean ± SEM; one-way ANOVA and Bonferroni’s post hoc comparison test were performed (*p<0.05, **p<0.005, ***p<0.0005, ****p<0.0001).

**Supplementary Figure S4. A** IHC for MAP2 (grey), mitochondrial marker TOMM20 (red) and LC3A (green) in primary neurons in vehicle and cells treated for 0.5h with CRH (scale bar = 5 μm) and relative quantification relative to soma. Experiments were performed in N=3 independent replicates at DIV14. Data are displayed as Mean ± SEM; one-way ANOVA and Bonferroni’s post hoc comparison test were performed (*p<0.05, **p<0.005, ***p<0.0005, ****p<0.0001).

**Supplementary Figure S5.** Analysis of mitochondrial fission *in vivo* **A** WB analysis and quantification of hippocampal homogenates using antibody directed against the mitochondrial fission marker DRP1^S616^ in Sham and in mice 5 days after TxT. Experiments were performed in N=3 independent replicates at DIV14. Data are displayed as Mean ± SEM; one-way ANOVA and Bonferroni’s post hoc comparison test were performed (*p<0.05, **p<0.005, ***p<0.0005, ****p<0.0001).

**Supplementary Figure S6.** Analysis of proinflammatory markers in primary hippocampal neurons: IL-6 and IL-17, **A-B** *in vitro* (vehicle, CRH 100nM for 0.5h and with CRH 0.5h + CRHR1 Blocker NBI30775 100nM) and **D-E** *in vivo* from hippocampal homogenates of Sham and of mice 5 days after TxT (5 TxT)**.** Experiments were performed in N=3 independent replicates at DIV14. Data are displayed as Mean ± SEM; one-way ANOVA and Bonferroni’s post hoc comparison test were performed (*p<0.05, **p<0.005, ***p<0.0005, ****p<0.0001).
